# Neuroanatomical mapping of huntingtin-associated protein 1 across the rostral and caudal clusters of mouse raphe nuclei and its immunohistochemical relationships with serotonin

**DOI:** 10.3389/fnana.2025.1625793

**Published:** 2025-07-22

**Authors:** Marya Afrin, Md Nabiul Islam, Mirza Mienur Meher, Mir Rubayet Jahan, Kanako Nozaki, Koh-hei Masumoto, Akie Yanai, Koh Shinoda

**Affiliations:** ^1^Division of Neuroanatomy, Department of Neuroscience, Yamaguchi University Graduate School of Medicine, Yamaguchi, Japan; ^2^Department of Anatomy and Histology, Faculty of Veterinary Science, Bangladesh Agricultural University, Mymensingh, Bangladesh; ^3^Department of Basic Laboratory Sciences, Faculty of Medicine and Health Sciences, Yamaguchi University Graduate School of Medicine, Yamaguchi, Japan; ^4^Department of Microbiology and Public Health, Faculty of Veterinary Medicine and Animal Science, Gazipur Agricultural University, Gazipur, Bangladesh; ^5^Department of Neurosurgery, Yamaguchi University Graduate School of Medicine, Yamaguchi, Japan; ^6^School of Human Care Studies, Nagoya University of Arts and Sciences, Nisshin, Aichi, Japan

**Keywords:** HAP1, stigmoid body, serotonergic neuron, neurodegenerative disorder, neuroprotection

## Abstract

Huntingtin-associated protein 1 (HAP1) is a crucial component of the stigmoid body (STB) and is recognized as a neuroprotective interactor with causative proteins for several neurodegenerative disorders (NDs). Due to HAP1 protectivity, brain regions rich in STB/HAP1 are typically shielded from neurodegeneration, whereas areas with little or no STB/HAP1 are often affected in NDs. Mounting evidence suggests that serotonin (5-HT) neuron dysfunction contributes to various NDs. While the raphe nuclei denote the origin of 5-HT neurons, HAP1 protectivity has yet to be determined there. To accomplish this, the present study evaluated the expression and detailed neuroanatomical distribution of HAP1 throughout the rostral and caudal clusters of raphe nuclei in adult mice brains and their morphological relationships with 5-HT by employing Western blotting and immunohistochemistry. Our results indicated that in the rostral cluster, HAP1-ir cells were extensively distributed across the caudal linear raphe, median raphe, dorsal raphe, supralemniscal raphe, caudal part of the dorsal raphe, pre-pontine and pontine raphe nuclei. In the caudal cluster, HAP1-ir neurons were disseminated throughout the raphe magnus, raphe obscurus, raphe pallidus, parapyramidal, and raphe interpositus nuclei. Our double-immunofluorescence labeling results confirmed that most of the 5-HT neurons contained HAP1 immunoreactivity throughout the rostral and caudal clusters of the raphe nuclei. These suggest that HAP1 is crucial for modulating/protecting serotonergic functions, plausibly by upholding 5-HT neuronal plasticity/integrity by raising the threshold for neurodegeneration. Our current findings might provide a fundamental basis for further research aimed at elucidating the role of STB/HAP1 in the pathophysiology of serotonin neurons.

## 1 Introduction

Huntingtin-associated protein 1 (HAP1) is considered a neural interactor of huntingtin, a protein responsible for Huntington’s disease (HD) (Li et al., 1995). HAP1 is principally enriched in the neurons of the brain, spinal cord, and enteric nervous system (Gutekunst et al., 1998; Li et al., 1998; Fujinaga et al., 2007, 2009; Islam et al., 2012, 2017; Tarif et al., 2021, 2023). HAP1 is most often expressed in the stigmoid body (STB), a physiological entity that is approximately 0.5–3 μm in diameter and considered a spherical to oval-shaped, non-membrane-bound cytoplasmic inclusion (Shinoda et al., 1992, 1993). Therefore, HAP1 has been known as an essential component and the determinant marker of the STB.

The rodent brain contains two spliced HAP1 isoforms, HAP1A and HAP1B, each with a distinct C-terminal sequence (Li et al., 1998; Fujinaga et al., 2007). While transfection of HAP1B-cDNA can lead to diffuse cytoplasmic expression, transfection of HAP1A-cDNA can induce the formation of cytoplasmic STB in cultured cells. Furthermore, several fusions of tiny STBs cause the HAP1-induced STB to enlarge (Fujinaga et al., 2007). Interestingly, when co-expressed with HAP1A, HAP1B is linked to HAP1A and sequestered to STBs from the early occurrence of small STB particles (Fujinaga et al., 2007). Accordingly, it has been hypothesized that the HAP1A/HAP1B expression ratio controls the number of STBs that form or the size of the STBs that expand (Li et al., 1998; Fujinaga et al., 2007).

It is postulated that STB/HAP1 acts as a protective barrier against apoptosis and cell death by raising the threshold of susceptibility to neurodegeneration and providing enhanced stability to neurons in HD or several other neurodegenerative disorders (NDs), such as spinal and bulbar muscular atrophy, spinocerebellar ataxia type 3 (SCA3), SCA17, and Joubert syndrome (Li et al., 2003; Fujinaga et al., 2004; Takeshita et al., 2006, 2011; Metzger et al., 2008; Islam et al., 2017). The striatum, thalamus, cerebral neocortex, cerebellum, and motor nuclei are considered neurodegenerative targets in various NDs, perhaps due to low or absent STB/HAP1 expression in these brain and spinal cord areas. Furthermore, it has been reported that the hypothalamus of HAP1 knockout mice brains is more predisposed to apoptosis/neurodegeneration (Li et al., 2003). However, in STB/HAP1-enriched brain areas such as the preoptic area, medial amygdala, and hypothalamic regions, NDs usually do not result in cell death (Fujinaga et al., 2004, 2009; Islam et al., 2017, 2022). These results led to the idea called the “HAP1 protection hypothesis” against neurodegeneration (Fujinaga et al., 2004, 2009; Islam et al., 2017, 2022, 2025; Wroblewski et al., 2018; Tarif et al., 2021, 2023).

Previous research has demonstrated that, in terms of physiological functions, hypothalamic HAP1 can regulate eating behavior and postnatal development (Chan, 2002; Li et al., 2003; Sheng et al., 2006; Lin et al., 2010; Xiang et al., 2014). The serotonin (5-hydroxytryptamine, 5-HT) can act as a growth factor during embryogenesis, and 5-HT receptor activation is a critical component of the series of events that lead to periodic changes in brain structure. Impaired brain development has been linked to CNS disorders, which may also occur or be exacerbated due to disruption of the 5-HT system (Sodhi and Sanders-Bush, 2004). Moreover, 5-HT neurons that are harbored within distinct subregions of raphe nuclei have unique electrophysiological, morphological, and receptor profiles that can be activated by stressors and could render them selectively sensitive to stress (Lukkes et al., 2011; Soiza-Reilly and Commons, 2011). Intriguingly, HAP1 can control hypothalamic glucocorticoid receptors at the protein level to alter the hypothalamic function related to stress response (Chen et al., 2020). Thus, it is possible that HAP1 might be involved in modulating the functions of 5-HT in raphe nuclei.

There is mounting evidence that the dysfunction of the 5-HT system is responsible for certain NDs (Meltzer et al., 1998; Fox et al., 2009; Huot et al., 2011; Politis and Niccolini, 2015). It might be possible that, owing to its putative protective properties, HAP1 can protect the 5-HT system against neurodegenerative stresses. Thus, revealing the relationships of HAP1 with 5-HT throughout the raphe nuclei is essential. Though the expression of HAP1 was cursorily described in rodent brainstem (Fujinaga et al., 2004; Islam et al., 2012), to the best of our knowledge, the expression and detailed neuroanatomical distribution of HAP1 have remained undefined throughout the rostral and caudal clusters of raphe nuclei. In this context, we attempted to elucidate the cytoarchitectonic boundary along with the comparative distribution of HAP1 and 5-HT in the raphe nuclei of adult mice. Here, we further made an effort to clarify the immunohistochemical relationship of HAP1 with 5HT in the raphe nuclei throughout the adult mouse brainstem (midbrain, pons, and medulla oblongata) using Western blotting and immunohistochemistry.

## 2 Materials and methods

### 2.1 Animals

In the present study, we used male C57BL/6J mice (8 weeks old; Japan SLC Inc., Shizuoka, Japan). The animals were housed (3–4 mice/cage) at 22°C constant temperature, kept in 12 h light–dark cycles (lights on 08:00–20:00) with free access to food and water independent of the diet. All efforts were made to reduce the number of mice used and to minimize animal suffering. The entire experimental protocol conformed to the guidelines for animal research by the Japanese government (Law No. 105, Notification No. 6) and was approved by the Committee on the Ethics of Animal Experimentation at Yamaguchi University School of Medicine.

### 2.2 Characterization of primary antibodies

**Figure 1:**
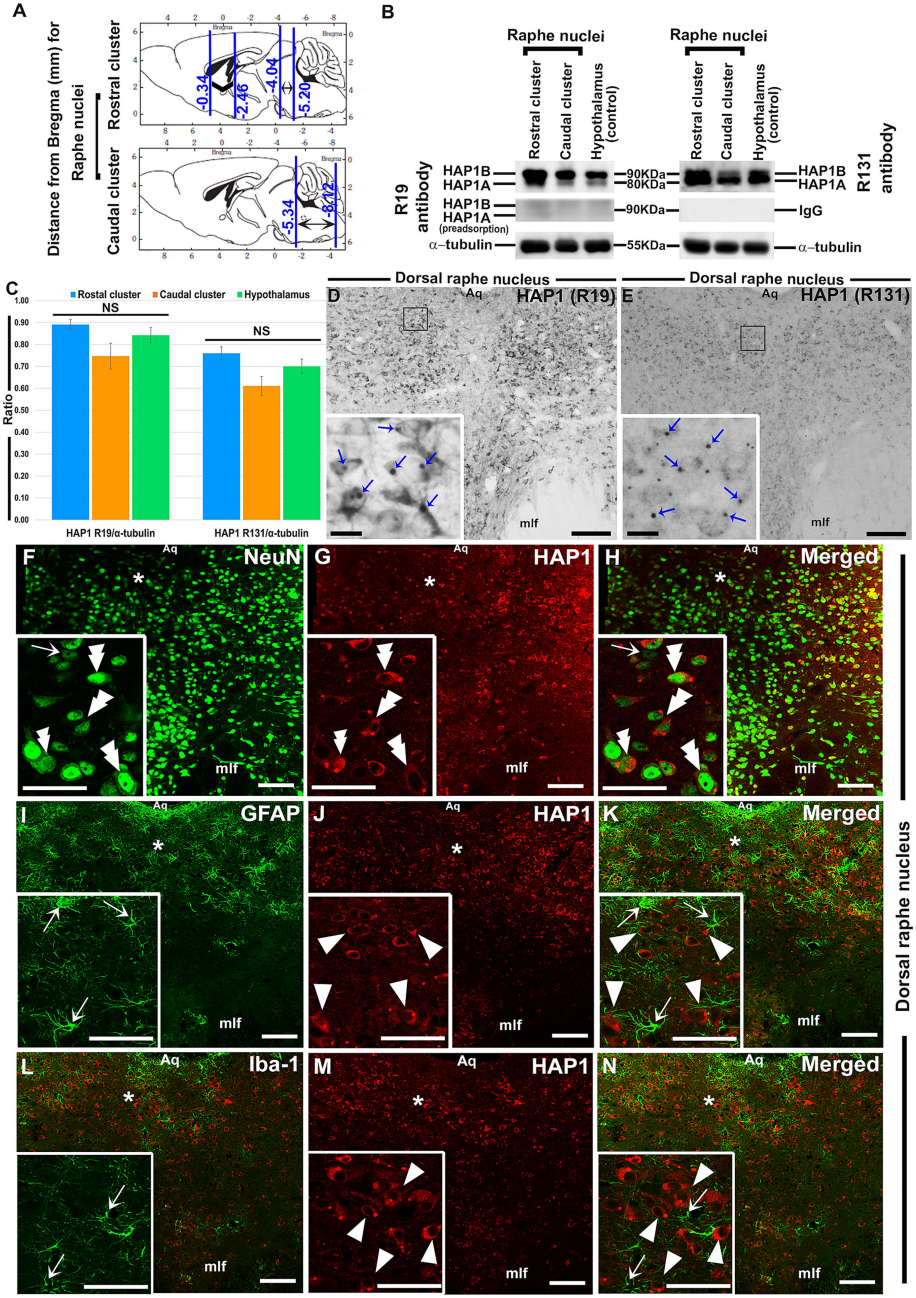
Western blotting and immunoperoxidase immunohistochemistry for HAP1 and double-label immunofluorescence immunohistochemistry for HAP1 with neuronal or glial cell markers. **(A)** Brain sections were examined for rostral (Bregma −4.04 to −5.20 mm) and caudal (Bregma −5.34 to −8.12 mm) clusters of raphe nuclei. **(B)** Western blot analysis showing the HAP1 R19 and R131 immunoreactive (ir) bands; Pre-adsorption of the anti-HAP1 R19 antibody or using normal rabbit IgG resulted in the disappearance of HAP1-ir bands. **(C)** The ratio (mean) of HAP1 R19 and HAP1 R131-ir bands to *α*-tubulin on the Western blots (*n* = 6). NS = non-significant. **(D,E)** Immunoperoxidase staining shows HAP1 immunoreactivity using R19 and R131. Blue arrows indicate the HAP1-ir stigmoid body (STB) in the insets of **(D,E)**. Photomicrograph showing double-label immunofluorescence staining of HAP1 and NeuN **(F–H)**, GFAP **(J–K)**, and Iba1 **(L–N)**, in which insets are the enlargement of respective areas indicated by asterisks. Double arrowheads indicate the cells positive for both HAP1 and NeuN. Single white arrowheads indicate cells single-positive for HAP1. White arrows indicate cells single-positive for NeuN, GFAP, or Iba1. Scale bar = 20 μm in **(D–N)** and insets. For abbreviations, see “List of Glossary”.

The sources, immunogens, and working dilutions of primary antibodies used in the present study are summarized in Table 1. To explore the complete pattern and localization of HAP1, we used two different polyclonal antibodies against HAP1 in immunoperoxidase staining. One is a commercially produced goat polyclonal anti-HAP1 antibody (R19), and the other is our laboratory-raised rabbit polyclonal anti-HAP1 antibody (R131) (Table 1). Our prior research has confirmed the specificity of these two antibodies (Fujinaga et al., 2007; Islam et al., 2017, 2020a, 2020b, 2022). We have also evaluated the antibody’s specificity in brainstem raphe nuclei using Western blotting (Figure 1B) and immunoperoxidase staining (Supplementary Figure 1) in this study. In addition, a commercially produced anti-5-HT primary antibody was used here, and its specificity was demonstrated in our previous studies (Table 1). We have also assessed the specificity of this 5-HT antibody employing immunoperoxidase staining (Supplementary Figure 1) in the present study. 5-HT is a small molecule with a molar mass of about 0.176 kDa. In this study, we could not detect the 5-HT band using Western blot analysis. Prior research had established the specificity of the other commercially available antibodies employed in this study, most of which were selected from the Neuroscience Information Framework’s Antibody Registry (Table 1).

**Table 1:** Detailed information on primary antibodies used in the present study.

Antibody	Immunogen	Host/antibody type	Catalog number/research resource identifiers (RRIDs)	Source	Dilution	References
Anti-HAP1 (R19)	Rat HAP1 C-terminus	Goat polyclonal	Cat# sc-8770 RRID: AB_647322	Santa Cruz Biotechnology, Santa Cruz, CA, USA	1:20,000	Islam et al. (2017, 2025)
Anti-HAP1 (R131)	GST-fused rat HAP1^70-433^	Rabbit polyclonal	RRID: AB_2571562	Div. of Neuroanatomy, Yamaguchi University	1:20,000	Fujinaga et al. (2007)
Anti-Serotonin (5-HT)	Serotonin covalently bound to bovine thyroglobulin with carbodiimide	Rabbit polyclonal	Cat# 066D, RRID: AB_2313879	Biomeda Corp. USA	1:5,000	Yanai et al. (2020) and Sheng et al. (2004)
Anti-GFAP	Purified human brain GFAP	Rabbit polyclonal	Cat# G9269, RRID: AB_477035	Sigma-Aldrich, St. Louis, MO, USA	1:1,000	Islam et al. (2012)
Anti-Iba1	Synthetic peptide corresponding to the C-terminus of Iba1	Rabbit polyclonal	Cat# 019–19741, RRID: AB_839504	Wako, Osaka, Japan	1:1,000	Wroblewski et al. (2018)
Anti-NeuN	Synthetic peptide of Human NeuN aa 1–100	Rabbit monoclonal	Cat# ab177487, RRID: AB_2532109	Abcam, Cambridge, UK	1:5,000	Islam et al. (2020a)
Anti-α tubulin	Microtubule derived from chicken embryonic brain	Mouse monoclonal	Cat# T6199, RRID: AB_477583	Sigma-Aldrich, St. Louis, MO, USA	1:200,000	Islam et al. (2021) and Tarif et al. (2023)

GFAP, glial fibrillary acidic protein; HAP1, Huntingtin-associated protein-1; Iba1, ionized calcium binding adaptor molecule 1; NeuN, neuronal nuclei.

### 2.3 Western blotting

Western blotting was accomplished as described in our previous studies (Islam et al., 2017, 2020a, 2020b). In brief, mouse brains (*n* = 6) were harvested immediately after transcardial perfusion with ice-cold saline. After that, each brain was positioned into the stainless-steel mouse brain matrix (1 mm) with the hypothalamus facing up. Then, the brains were sectioned by placing chilled microtome blades along the extension of the hypothalamus (bregma, −0.34 to −2.46 mm), the rostral cluster (bregma, −4.04 to −5.20 mm), and the caudal cluster (bregma, −5.34 to −8.12 mm) of the brainstem raphe nuclei (Figure 1A). The hypothalamus (control) from the left and right sides of each individual mouse brain, as well as the rostral and caudal clusters of the raphe nuclei that are dispersed along the midline of the brainstem, were collected separately by micropunching under the microscope. Then the collected tissues were homogenized in tissue protein extraction reagent (T-PER; Thermo Scientific, Rockford, IL, United States) added with 5 μL/mL of protease inhibitor (P8340; Sigma–Aldrich). The protein concentration for each sample was ascertained through the BCA Protein Assay method (Thermo Scientific, Waltham, MA, United States). Eighty microgram of each protein was loaded, separated by 7.5% SDS-polyacrylamide gel electrophoresis, and then transferred onto a polyvinylidene difluoride membrane utilizing a wet transfer apparatus. Membranes were blocked for 2 h with 5% skimmed milk in Tris-buffered saline with 0.1% Tween-20 (TBST) and probed overnight with the primary antibodies: goat polyclonal anti-HAP1 (R19) (1:20,000), rabbit polyclonal anti-HAP1 (R131) (1:20,000), rabbit polyclonal anti-IgG (1:5000) or mouse monoclonal anti-*α* tubulin (1:200,000) (Table 1). To perform the HAP1 (R19) preadsorption test, the diluted antibody was treated with a particular blocking peptide (sc-8770P, Santa Cruz Biotechnology, Santa Cruz, CA, United States) overnight at 4°C. Following several washes with TBST, the membrane was treated with horseradish peroxidase-conjugated secondary antibody: anti-goat (1:10,000), anti-rabbit (1:5,000), or anti-mouse (1:20,000) antibody for 2 h at room temperature (Table 2). Enhanced chemiluminescence reagents (ECL select; GE Healthcare) and an Amersham Imager 600 (GE Healthcare) were used to visualize immunopositive protein bands after repeated washes in TBST. Densitometric analysis was conducted to quantify the immunoreactive band ratio of HAP1 R19 and R131 to α-tubulin between the rostral and caudal clusters using ImageJ software (NIH, Bethesda, MD, United States).

**Table 2:** Detailed information on secondary antibodies and other reagents used in the present study.

Secondary antibody and other reagents	Immunogen	Host	Catalog number/research resource identifiers (RRIDs)/source	Application	Dilution	References
HRP-labeled anti-goat	Goat IgG, F(ab’) 2	Donkey	Cat# 3851, RRID: AB_641200 Santa Cruz Biotechnology, Santa Cruz, CA, USA.	Western blotting	1:10,000	Islam et al. (2022, 2025)
HRP-labeled anti-rabbit	Rabbit IgG F(ab’) 2	Donkey	Cat# NA934, RRID: AB_772206 Cytiva, Sigma-Aldrich, Germany.	Western blotting	1:5,000	Islam et al. (2017)
HRP-labeled anti-mouse	Mouse IgG whole molecule	Sheep	Cat# NA931, RRID: AB_772210 GE Healthcare, Buckinghamshire, UK.	Western blotting	1:20,000	Islam et al. (2012)
Biotinylated donkey anti-goat	IgG, isolated from goat serum	Donkey	Cat# AP180B, RRID: AB_92569 Millipore, Darmstadt, Germany.	Immunoperoxidase staining	1:1,000	Jahan et al. (2015)
Biotinylated goat anti-rabbit	IgG, isolated from rabbit serum	Goat	Cat# AP180B, RRID: AB_92569 Millipore, Darmstadt, Germany.	Immunoperoxidase staining	1:1,000	Islam et al. (2017, 2021)
Alexa Fluor 594 donkey anti-goat	IgG, isolated from goat serum	Donkey	Cat# A-11058, RRID: AB_2534105 Invitrogen, Eugene, OR, USA.	Immunofluorescence staining	1:1,000	Tarif et al. (2023)
Alexa Fluor 488 donkey anti-rabbit	IgG, isolated from rabbit serum	Donkey	Cat# A32790, RRID: AB_2762833 Invitrogen, Eugene, OR, USA.	Immunofluorescence staining	1:1,000	Islam et al. (2020a)
HRP-conjugated streptavidin	N/A	N/A	Cat# P0397, Agilent Dako, Glostrup, Denmark.	Immunoperoxidase staining	1:1,000	Yanai et al. (2020)

HRP, horseradish peroxidase; IgG, immunoglobulin G.

### 2.4 Tissue preparation for immunohistochemistry

The mice were first anesthetized with an anesthetic mixture (MMB; Kirihara et al., 2012) of medetomidine (Domitor^®^, Orion Pharma, Espoo, Finland; 0.3 mg/kg), midazolam (Midazolam^®^, Sandoz, Basel, Switzerland; 4.0 mg/kg), and butorphanol (Vetorphale^®^, Meiji Seika Pharma, Tokyo, Japan; 5.0 mg/kg). Each mouse was injected with 0.25–0.30 mL of MMB intraperitoneally and then transcardially perfused with ice-cold saline, followed by 4% paraformaldehyde in 0.1 M phosphate buffer (PB; pH 7.4). After removing the brain, the same fixative used for perfusion was employed to postfix for 24 h. Then, the brain was cryoprotected with immersion in 30% sucrose in 0.1 M PB until it sank. The brainstem (midbrain, pons, and medulla oblongata) was sectioned serially from bregma −4.04 to −8.12 mm in the coronal plane at 30 μm intervals (Figure 1B) on a freezing microtome (Leica CM1900). After that, frozen sections were kept at 4°C in an ice-cold 0.02 M sodium phosphate-buffered solution (PBS; pH 7.4) with 0.1% sodium azide until used for immunohistochemistry.

### 2.5 Immunoperoxidase staining

Free-floating immunohistochemistry was performed using the brainstem (midbrain, pons, and medulla oblongata) section as previously described (Islam et al., 2012, 2017). Sections were first blocked and permeabilized in PBS containing 10% normal donkey serum (NDS) or 10% normal goat serum (NGS) and 0.3% triton X-100 (TX) for 2 h. Following this, sections were pretreated with 50% cold methanol and 1.5% H_2_O_2_ at 4°C for 30 min. Sections were then incubated with primary antibodies to HAP1 R19 and R131 (1:20,000) or rabbit polyclonal anti-IgG (1:5000) for 5 days at room temperature or to 5-HT (1:5,000) at 37°C for 7 days. The blocking peptide (sc-8770P, Santa Cruz Biotechnology, Santa Cruz, CA, United States) was added to the diluted primary antibody (R19) to conduct the preadsorption staining. Following primary antibody immunoreaction, sections were washed and then incubated overnight at 4°C in PBS containing 1% NDS or NGS and biotinylated donkey anti-goat or goat anti-rabbit secondary antibody (1:1,000). This was followed by a 2 h incubation at 20°C with peroxidase-conjugated streptavidin (Dako, Glostrup, Denmark; 1:1000 diluted in PBS). Details on antibodies and selected reagents are included in Tables 1, 2. The sections were then washed in 0.05 M Tris–HCl buffer (pH 7.6) followed by a violet-to-black color reaction using 0.02% 3,3’diaminobenzidine (DAB; Wako Pure Chemical Industries, Ltd., Osaka, Japan) and 0.6% nickel ammonium sulfate (Sigma–Aldrich, Tokyo, Japan) in 0.05 M Tris–HCl buffer containing 0.0008% hydrogen peroxide (nickel-enhanced DAB reaction) for approximately 10 min on ice. Sections were mounted on glass slides, dehydrated in ethanol following air drying, cleared in xylene, and coverslipped using Entellan Neu (Merck KGaA, Darmstadt, Germany).

### 2.6 Immunofluorescence staining

Double-label immunofluorescence staining was carried out in accordance with our earlier research (Islam et al., 2012, 2017, 2023; Yanai et al., 2020), and the list of antibodies and particular reagents were included in Tables 1, 2. For immunofluorescent staining, brainstem sections were blocked with PBS containing 10% NDS and 0.3% Triton X-100 for 2 h at 4°C. Sections were then washed, followed by incubation with goat anti-HAP1 antibody (R19, 1:6000) and rabbit anti-5-HT (1:2000) for 7 days at 37°C or goat anti-HAP1 antibody (R19, 1:10000) in combination with rabbit anti-neuronal nuclei (NeuN) (1:5000), rabbit anti-GFAP (1:1000) or rabbit anti-Iba1 (1:1000) in PBS containing 1% NDS and 0.3% Triton X-100 for 5 days at 20°C. After repeated washing, sections were incubated with a mixture of Alexa Fluor 594-conjugated donkey anti-goat and Alexa Fluor 488-conjugated donkey anti-rabbit IgG (Molecular Probes, Eugene, OR, United States; 1:1000 diluted in PBS containing 1% NDS) at 20°C for 2 h. After that, sections were washed with PBS, mounted on glass slides, air-dried, and embedded with Fluoromount Plus (K048, Diagnostic Biosystems, Pleasanton, CA, United States). Before being used, slides were kept at 4°C in a light-tight slide box.

### 2.7 Cytoarchitectonic analysis and terminology

The cytoarchitectonic assessment was completed using Nissl staining (cresyl violet; Merck) of sections neighboring or adjoining those stained for 5-HT or HAP1. Along the midline of the brainstem, raphe nuclei are allocated based on their distribution into two clusters: the rostral cluster, confined to the mesencephalon (caudal midbrain) and rostral pons, and the caudal cluster, extending from the caudal pons to the caudal portion of the medulla oblongata (Alonso et al., 2013). The center part of the brainstem has been separated into two zones: the median zone and the paramedian zone, within which raphe nuclei are included (Alonso et al., 2013). Additionally, the hindbrain and a portion of the midbrain are temporarily split into many areas known as rhombomeres. The rostral cluster of raphe nuclei is generated by rhombomeres 1, 2, and 3, whereas the caudal raphe cluster is generated by rhombomeres 5–7 (Weissbourd et al., 2014). Nomenclature for the entire raphe nuclei area in the mouse brain was confirmed by previous research and subsequently adopted by the mouse brain atlas (Paxinos and Franklin, 2008), which was followed in our current study.

### 2.8 Cell counting

For cell counting, 40x objective immunofluorescence images were taken and imported into ImageJ software (NIH, Bethesda, MD, United States). The actual number of HAP1 or 5-HT-immunoreactive (ir) neurons and those double-stained for HAP1 and 5-HT were used to compute co-expression ratios for HAP1 in 5-HT, or 5-HT in HAP1 (Table 3), in accordance with the counting protocol outlined in our earlier study (Nagano and Shinoda, 1994; Jahan et al., 2015). TN = N × D/{(T + W) × S} [TN, total number] of 5-HT in HAP1-ir cells or HAP1 in 5-HT- ir cells in individual raphe nuclei of rostral and caudal clusters; N, 5-HT or HAP1-ir neurons along with HAP1 and 5-HT double-stained neurons observed in sections; D, distance between the first and last sections in which raphe nuclei were counted (μm); T, section thickness (30 μm in the present study); W, mean diameter of the raphe nuclei (8 μm for the mouse in the present study); S, number of sections in which distinct raphe nuclei were counted. Four sections from a single mouse were used for each nucleus, with no closure of 60 μm between sections. Six mice were utilized to count the rostral and caudal clusters of raphe nuclei.

**Table 3:** Co-expression ratios of HAP1/5-HT or 5-HT/HAP1 in the brainstem raphe nuclei of adult mice.

Brainstem area	Raphe nucleus		Mean ± SD		LS (student’s *t*-test)
			HAP1/5-HT co-expression ratio (%)	5-HT/HAP1 co-expression ratio (%)	
Midbrain	Rostral cluster	Median raphe (MnR)	95.56^d^ ± 0.76	70.45^g^ ± 0.6	**
		Paramedian raphe (PMnR)	HAP1/0^#^	0^#^/HAP1	N/A
		Supralemniscal raphe (SuL)	98.44^ab^ ± ±0.59	90.66^d^ ± 0.52	**
		Caudal linear raphe (CLi)	97.76^abc^ ± 0.66	97.78^a^ ± 0.36	NS
		Dorsal raphe dorsal (DRD)	90.61^e^ ± 1.46	82.59^f^ ± 0.73	**
		Dorsal raphe ventral (DRV)	96^cd^ ± 2.29	85.98^e^ ± 0.52	**
		Dorsal raphe interfascicular (DRI)	97.6^bc^ ± 0.71	94.73^bc^ ± 0.64	**
		Dorsal raphe lateral (DRL)	85.65^f^ ± 0.68	80.36^f^ ± 0.5	**
		Posterodorsal raphe (PDR)	98.45^ab^ ± 0.61	93.66^c^ ± 0.47	**
		Dorsal raphe caudal (DRC)	96.49^bcd^ ± 1.11	86.19^e^ ± 0.37	**
Rostral pons	
		Prepontine raphe (PPnR)	98.43^ab^ ± 0.85	96.02^abc^ ± 0.76	*
Pontine raphe (PnR)	97.84^abc^ ± 0.72	96.91^ab^ ± 0.45	NS
Caudal pons	Caudal cluster	Raphe magnus (RMg)	97.42^bcd^ ± 0.43	90.68^d^ ± 0.41	**
		Raphe interpositus (RIP)	0^#^/HAP1	HAP1/0^#^	N/A
		Raphe obscurus (ROb)	99.67^a^ ± 0.44	94.66^bc^ ± 0.91	**
		Raphe pallidus (RPa)	97.38^bcd^ ± 0.65	81.19^f^ ± 0.4	**
Medulla	
Parapyramidal raphe (PPy)	98.2^ab^ ± 1.52	88.96^d^ ± 0.48	**
LS (One way ANOVA followed by Tukey’s test)			**	**	

Values represent the mean ± SD of co-expression ratios (%) in adult mice (*n* = 6). ^a,b,c,d,e,f,g^Denote the means within the same column with different superscript letters that differ significantly. **Significant at 1% (*p* < 0.01); *significant at 5% (*p* < 0.05); ^#^no co-expression, 5-HT immunoreactivity was absent in both PMnR and RIP nuclei; N/A, not applicable; NS, non-significant; LS, level of significance; SD, standard deviation; 5-HT, serotonin; HAP1, Huntingtin-associated protein 1.

### 2.9 Statistical analysis

The Statistical Package for Social Sciences (SPSS), version 25.0, was used to examine every data set. Instead of performing a formal sample size calculation, for statistical analysis, the number of animals used (*n* = 6 mice for each analysis) was determined based on prior published studies in the brainstem (Islam et al., 2022). The design follows the three ‘R’ approach (replacement, reduction, refinement), focusing on minimizing animal usage while upholding scientific integrity and statistical rigor. For the computation and graphical representation of Western blot protein intensity, the relative band optical density ratio of HAP1 R19/*α*-tubulin or HAP1 R131/α-tubulin data was imported into MS Excel (Microsoft Office Excel 2019, United States). Subsequently, one-way Analysis of Variance (ANOVA) was conducted, followed by a post-hoc test (Tukey’s test) to compare the means using SPSS v. 25. Additionally, the other chosen sample size (*n* = 6) permitted parametric statistical analysis of HAP1/5-HT and 5-HT/HAP1 cell count ratios. The Student’s t-test was then used for pairwise comparisons of the HAP1/5-HT and 5-HT/HAP1 cell count ratios, and the one-way ANOVA was followed by Tukey’s *post hoc* test for regional comparisons. For every test, *p*-values <0.01 or <0.05 were deemed significant. To verify inter-animal variability, the statistical unit used in our data is the average co-expression ratio per animal (*n* = 6), which is derived from numerous sections for each raphe nucleus.

### 2.10 Photomicrographs

For light microscopic images, a color digital Lumenera USB 2.0 camera (Lumenera Corporation, Ottawa, Canada) equipped with an Eclipse E80i photomicroscope (Nikon) and image tiling software (Mitani Corporation, Tokyo, Japan) was used to take photomicrographs. For fluorescence images, a confocal laser microscope (Leica Microsystems, STELLARIS 8; Wetzlar, Germany) with LAS X software was used to attain single optical sections (4,296 × 4,296 pixels). The original photographs were left unaltered, with the exception of adjusting the brightness and contrast using Adobe Photoshop CS 8.0 (Adobe Systems, Inc. San Jose, CA, United States).

## 3 Results

### 3.1 General expression of HAP1 immunoreaction in the raphe nuclei

The expression of HAP1 in the raphe nuclei was examined in immunohistochemistry and Western blotting (Figures 1A–D). Both HAP1A and HAP1B isoforms (about ~90 kDa for HAP1B; ~80 kDa for HAP1A) were detected in the rostral and caudal clusters of mouse brainstem raphe nuclei (Figure 1B) in Western blotting using goat polyclonal anti-HAP1 (R19) or rabbit polyclonal anti-HAP1 (R131) antibody. The immunoreactivity was substantially abolished after using a specific blocking peptide against the anti-HAP1 (R19) primary antibody or using normal rabbit IgG as a negative control against the HAP1 (R131) antibody (Figure 1B). The protein band ratio of HAP1 R19 and R131 to α-tubulin (Figure 1C) was used to quantitatively analyze the relative strength of HAP1 immunoreactivity in both clusters of raphe nuclei, yielding results indicative of at least six separate analyses. Our results showed that HAP1 immunoreactivity between the rostral and caudal clusters of the mouse brainstem raphe nuclei did not vary significantly (*p* > 0.05) using both HAP1 R19 and HAP1 R131 antibodies (Figure 1C). In immunohistochemistry, both R19 and R131 antibodies showed a similar expression pattern of HAP1 in the raphe nucleus (Figures 1D,E). Most HAP1-ir cells contained STBs in diffusely HAP1-ir cytoplasm, whereas few showed only diffuse cytoplasmic immunoreactivity for HAP1 with no clearly visible STBs (Figures 1D,E). These suggest that most HAP1-ir cells transcript and translate both HAP1A and HAP1B isoforms in the raphe nucleus. Smaller HAP1-ir STBs, however, were often masked by diffusely expanding intense HAP1 immunoreaction in the cytoplasm. Therefore, in immunohistochemistry, it was challenging in light microscopy to determine whether cytoplasmic diffuse HAP1 particles are unbound to the minor STB units.

Double-label immunohistochemistry for HAP1 and nuclear neuronal marker (NeuN), astrocyte marker (GFAP), or microglia marker (Iba1) was carried out to uncover the type of HAP1-ir cells in brainstem raphe nuclei. Significant NeuN immunoreactivity was seen in most of the HAP1-ir cells of the dorsal raphe nucleus (Figures 1F–H). On the other hand, GFAP (Figures 1I–K) and Iba1-ir cells (Figures 1L–N) never contained HAP1 immunoreactivity. These findings confirmed that HAP1-ir cells in the brainstem raphe nuclei shared no characteristics with glial cells, but they did show characteristics of neurons.

**Figure 2:**
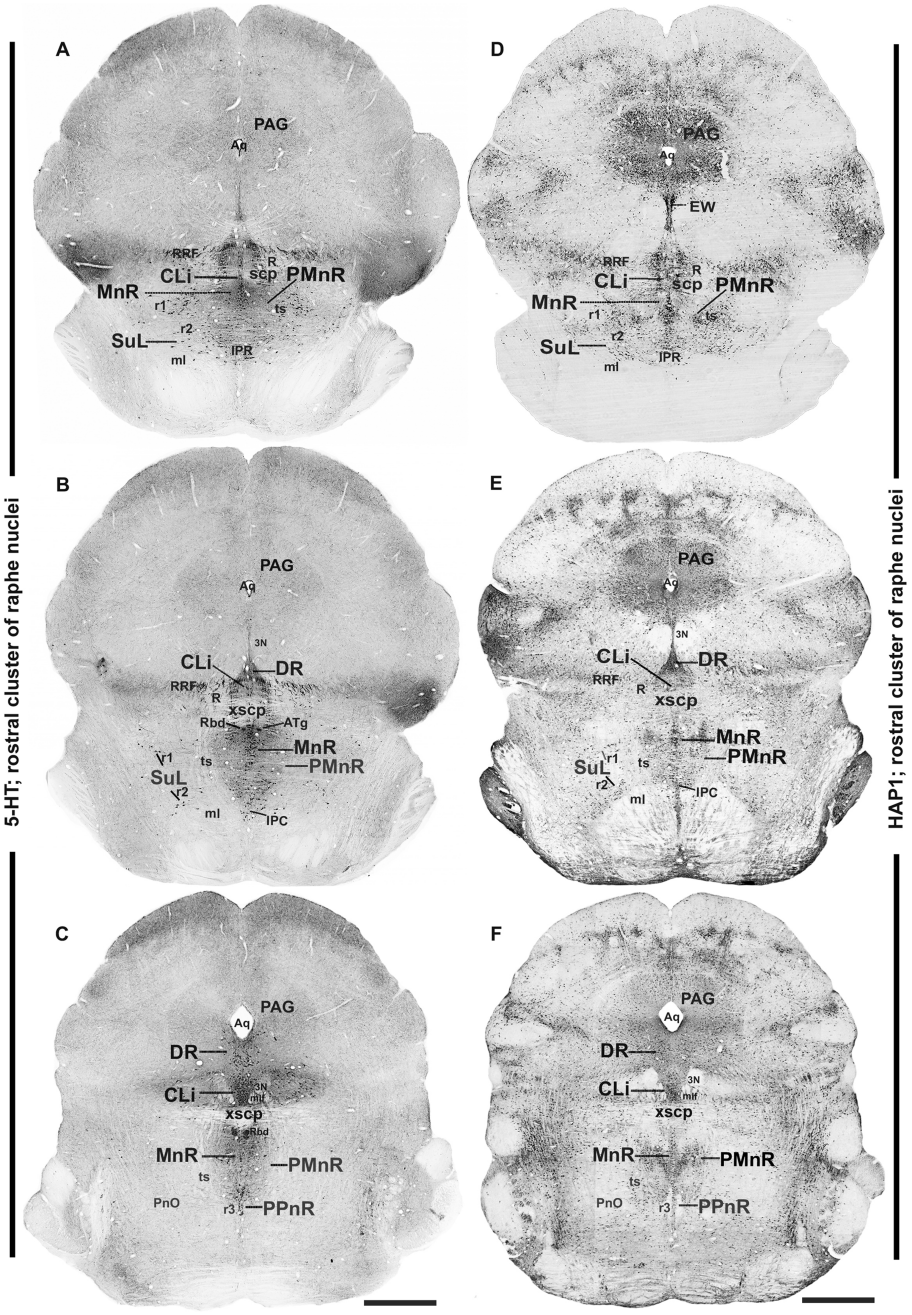
Distribution of 5-HT and HAP1 immunoreactivity across the caudal midbrain raphe nuclei (rostral cluster). Photomicrographs showing the 5-HT **(A–C)** and HAP1 **(D–F)** immunoreaction throughout the raphe nuclei in caudal midbrain. Selected structures are identified for topographic reference. Scale bar = 125 μm. For abbreviations, see “List of Glossary”.

**Figure 3:**
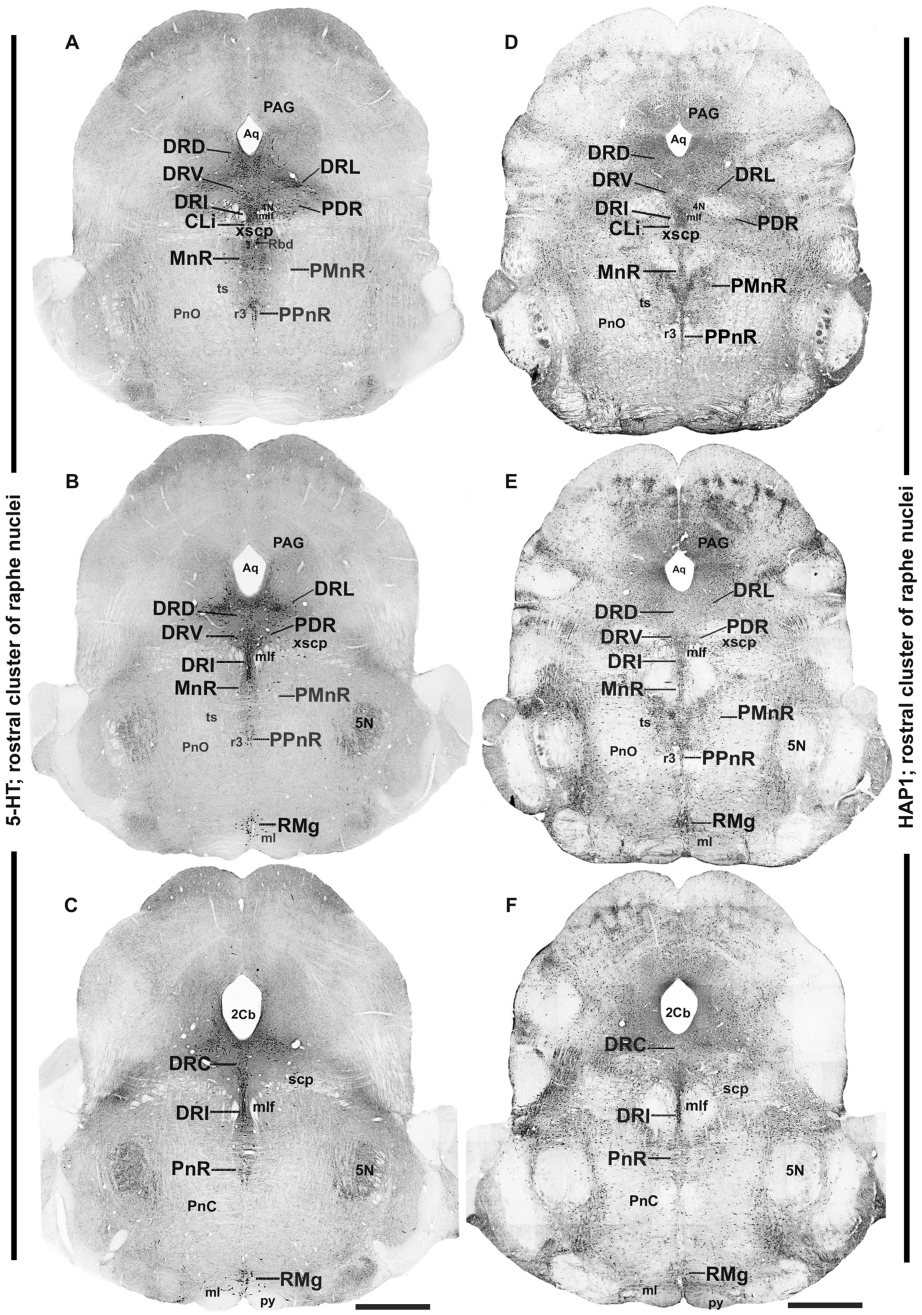
Distribution of 5-HT and HAP1 immunoreactivity across the rostral pons raphe nuclei (rostral cluster). Photomicrographs showing the 5-HT **(A–C)** and HAP1 **(D–F)** immunoreaction throughout the raphe nuclei in rostral pons. Scale bar = 125 μm. For abbreviations, “List of Glossary”.

**Figure 4:**
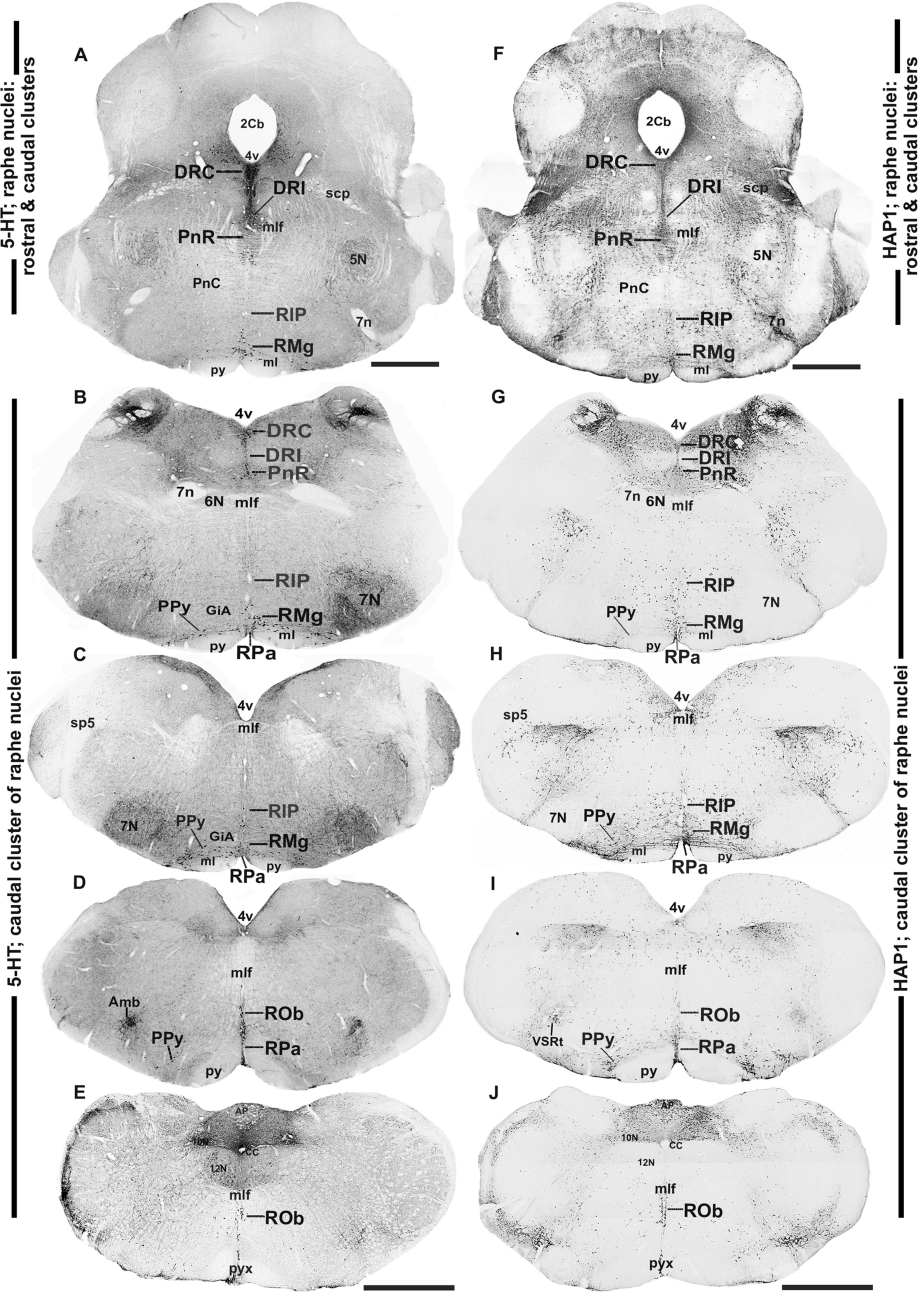
Distribution of 5-HT and HAP1 immunoreactivity across the caudal pons and medullary raphe nuclei (caudal cluster). Photomicrographs showing the 5-HT **(A–E)** and HAP1 **(F–J)** immunoreaction throughout the raphe nuclei in the caudal pons and medulla. Scale bar = 125 μm. For abbreviations, see “List of Glossary”.

Next, to examine the detailed distribution of HAP1-ir neurons in the rostral and caudal clusters of the raphe nuclei, we mapped the comparative neuroanatomical distribution of 5-HT and HAP1 using neighboring sections. The regional distribution of 5-HT (Figures 2A–C, 3A–C, 4A–E) and HAP1-ir cells (Figures 2D–F, 3D–F, 4F–J) was summarized in a non-consecutive series of maps throughout the rostral and caudal clusters of the raphe nuclei.

### 3.2 Distribution of HAP1-ir neurons in the rostral cluster of raphe nuclei: a comparison with 5-HT

**Figure 5:**
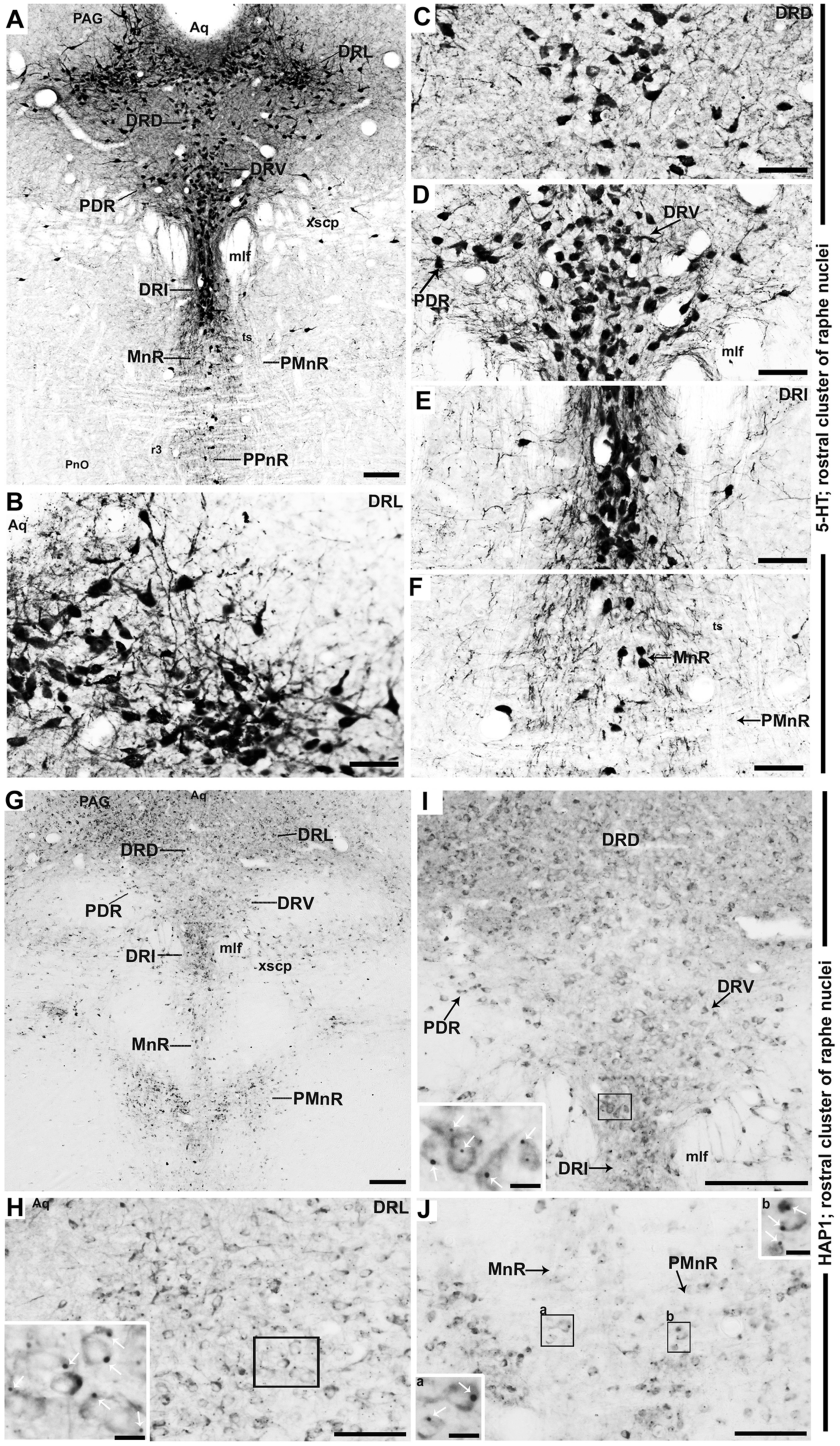
Selected high magnification immunoperoxidase images of 5-HT and HAP1 immunoreactivity in the rostral cluster of raphe nuclei. Photomicrographs showing the strong 5-HT **(A–F)** and HAP1-ir cells **(G–J)** in DRL, DRD, DRV, PDR, DRI, and MnR. The insets in **(H–J)** are the enlargement of the box areas, and the white arrows indicate STB. Scale bar = 120 μm in **(A)**; 100 μm in **(B–J)**; 20 μm in insets. For abbreviations, see “List of Glossary”.

In the rostral cluster of raphe nuclei, both 5-HT and HAP1-ir cells were highly distributed in the caudal linear nucleus (CLi), median raphe nucleus (MnR), dorsal raphe nucleus (DR), supralemniscal raphe nucleus (SuL), caudal part of dorsal raphe nucleus (DRC), prepontine raphe nucleus (PPnR), and pontine raphe nucleus (PnR) (Figures 2A–F, 3A–F, 4A,F). DR is the largest of all raphe nuclei. There are five subregions of DR, including the dorsal raphe nucleus dorsal part (DRD), dorsal raphe nucleus ventral part (DRV), dorsal raphe nucleus lateral part (DRL), posterodorsal raphe nucleus (PDR), and dorsal raphe nucleus interfascicular part (DRI). Strongly stained 5-HT (Figures 5A–E) and HAP1-ir neurons (Figures 5G–I) were uniformly disseminated in the DRD, DRV, DRI, DRL, and PDR. In the MnR, 5-HT cell bodies were distributed unevenly (Figures 5A,F). 5-HT neurons were more prevalent in the rostro-dorsal part of the MnR than in the ventro-caudal region. Moderately stained HAP1-ir neurons, however, were evenly distributed throughout the MnR (Figures 5G,J). Interestingly, 5-HT neurons appeared to be absent in the paramedian raphe nucleus (PMnR) (Figures 5A,F). In contrast, HAP1-ir neurons were strongly expressed in the PMnR (Figures 5G,J). Dot-like HAP1-ir STBs were observed in most of the raphe nuclei of the rostral cluster (figures in insets of Figures 5G–J).

### 3.3 Distribution of HAP1-ir neurons in the caudal cluster of raphe nuclei: a comparison with 5-HT

**Figure 6:**
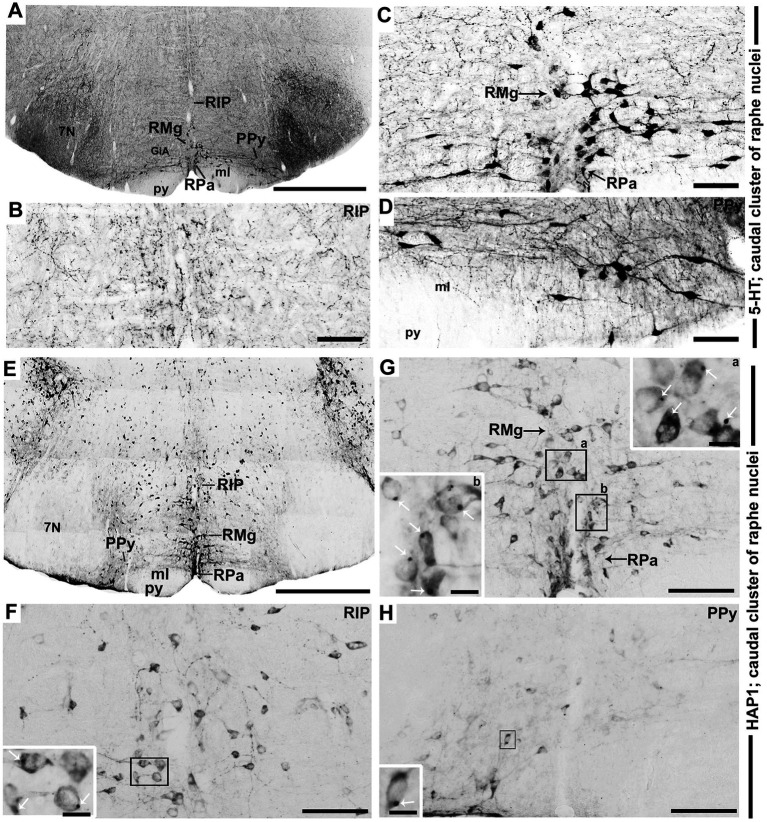
Selected high magnification immunoperoxidase images of 5-HT and HAP1 immunoreactivity in the caudal cluster of raphe nuclei. Photomicrographs showing the strong 5-HT **(A–D)** and HAP1-ir cells **(E–H)** in RIP, RMg, RPa, and PPy. The insets in **(F–H)** are the enlargement of the box areas, and the white arrows indicate STB. Scale bar = 50 μm in **(A,E)**; 100 μm in **(B-D and F-H)**; 20 μm in insets. For abbreviations see “List of Glossary”.

In the caudal cluster of raphe nuclei, both 5-HT (Figures 6A–D) and HAP1-ir (Figures 6E–H) cells were disseminated in the raphe magnus nucleus (RMg), raphe obscurus nucleus (ROb), and raphe pallidus nucleus (RPa) with its lateral expansion referred to as the medullary parapyramidal nucleus (PPy). Raphe interpositus (RIP) is a tiny nucleus in the brainstem that is located on the midline of the caudal pons. The RIP is located where the rootlets of the abducens nerve pass through the brainstem, lying beneath the mlf (Figures 4B,C,G,H). Intriguingly, the RIP was devoid of 5-HT neurons (Figures 6A,B), while intensely stained HAP1-ir neurons were detected in the RIP (Figures 6E,F). RMg is considered the most rostral and the largest of the caudal cluster. In RMg, 5-HT neurons were polygonal and perpendicularly oriented in close proximity to the midline (Figures 6A,C). 5-HT neurons seemed to be dispersed unevenly across the RMg nucleus (Figures 6A,C). Strongly stained HAP1-ir neurons were also disseminated in the RMg (Figures 6E,G). RPa is dispersed rostrocaudally throughout the paramedian basal plate of the caudal medulla oblongata (Figures 4B–D,G). A much smaller dispersion of the 5-HT neurons was observed in RPa (Figures 6A,C), while moderately stained HAP1-ir neurons were found there (Figures 6E,G). Additionally, the lateral expansion of RPa, referred to as PPy, encapsulated the pyramidal tract located laterally (Figures 4B–D,G–I). This PPy contained very scattered 5-HT neurons (Figures 6A,D). In contrast, HAP1 showed weak staining in the PPy, which sporadically extended toward the lateral side (Figures 6E,H). Dot-like HAP1-ir STBs were observed in most of the raphe nuclei of the caudal cluster (figures in insets of Figures 6F–H).

Our immunoperoxidase staining results revealed an almost similar distribution pattern of HAP1 and 5-HT across the rostral and caudal clusters of the raphe nuclei. To further investigate their immunohistochemical relationships, we subsequently conducted double-label immunofluorescent immunohistochemistry for HAP1 and 5-HT.

### 3.4 Immunohistochemical relationships of 5-HT and HAP1 in the rostral and caudal cluster

**Figure 7:**
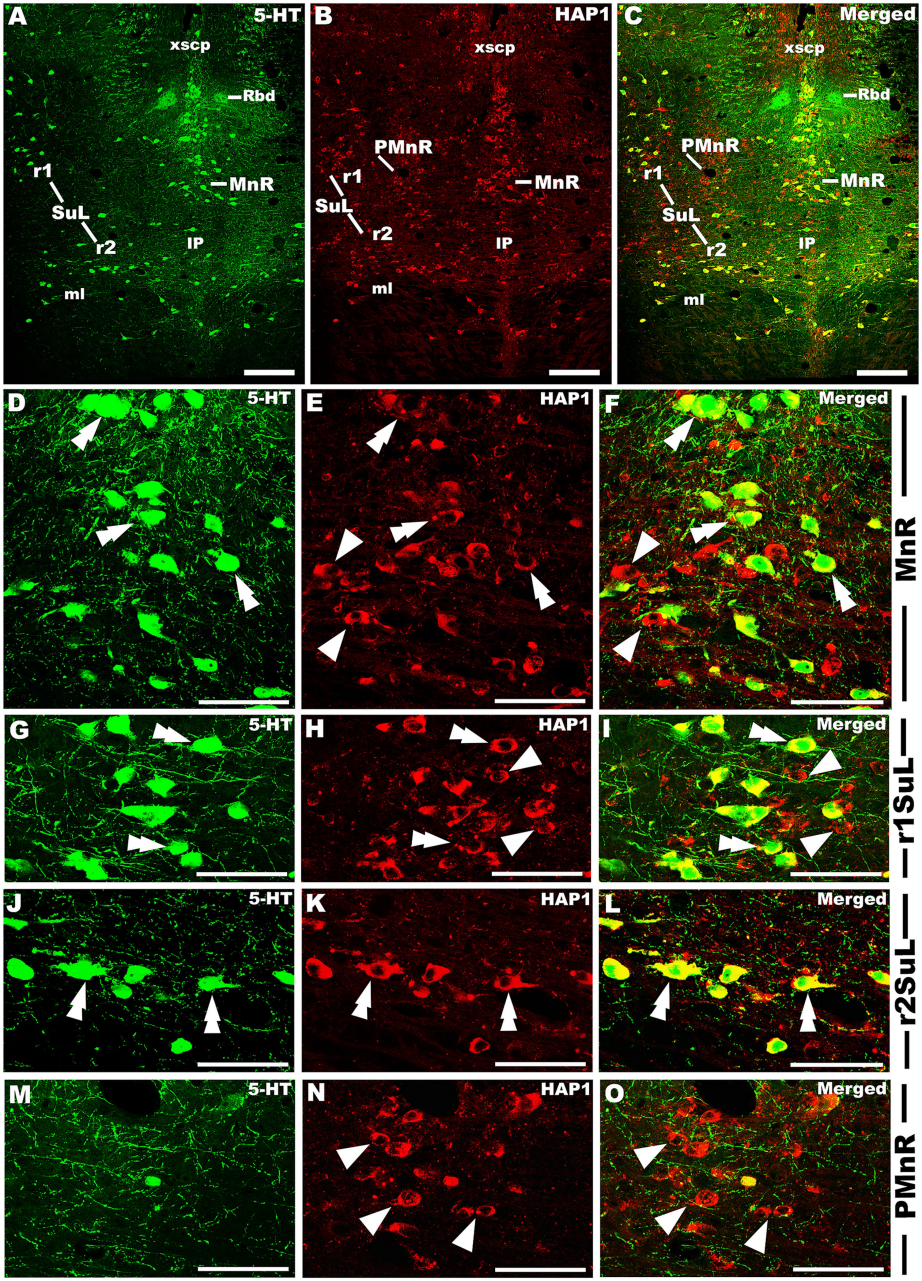
Double-label immunofluorescent immunohistochemistry for 5-HT and HAP1 in the median raphe nucleus and its lineage. Photomicrographs showing double-label immunofluorescence staining of 5-HT and HAP1 in MnR, PMnR, and SuL **(A–C)**. Panels **(D–O)** are the enlargement of MnR, r1SuL, r2SuL, and PMnR in **(A–C)**. Double arrowheads indicate the cells positive for HAP1 and 5-HT, white arrows and single white arrowheads indicate the cells positive only for 5-HT and HAP1, respectively. Scale bar = 20 μm in **(A–O)**. For abbreviations, see “List of Glossary”.

**Figure 8:**
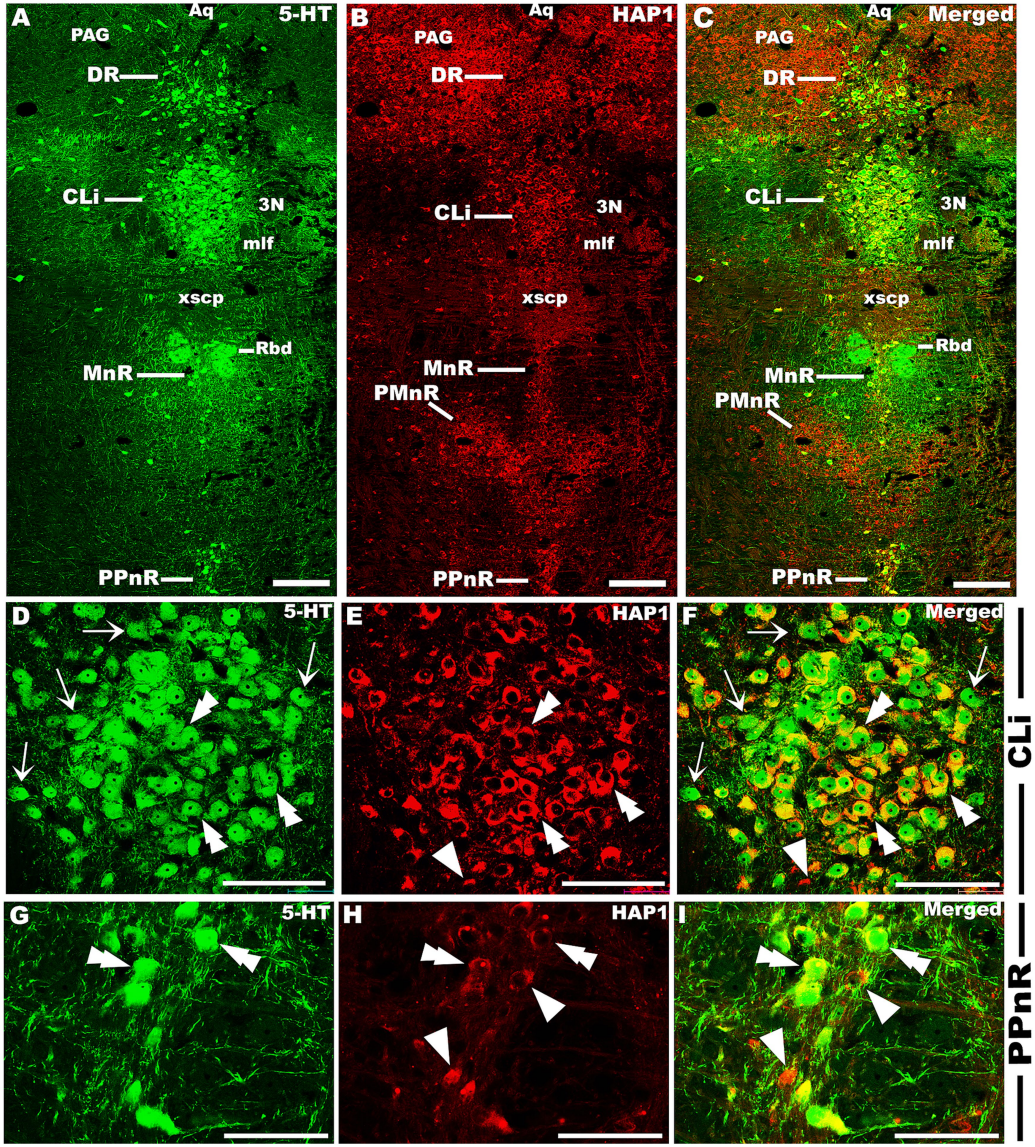
Double-label immunofluorescent immunohistochemistry for 5-HT and HAP1 in the caudal linear and prepontine raphe nuclei. Photomicrographs showing double-label immunofluorescence staining of 5-HT and HAP1 in CLi and PPnR **(A–C)**. Panels **(D–I)** are the enlargement of CLi and PPnR in **(A–C)**. Double arrowheads indicate the cells positive for HAP1 and 5-HT, white arrows and single white arrowheads indicate the cells positive only for 5-HT and HAP1, respectively. Scale bar = 40 μm in **(A–I)**. For abbreviations, see “List of Glossary”.

**Figure 9:**
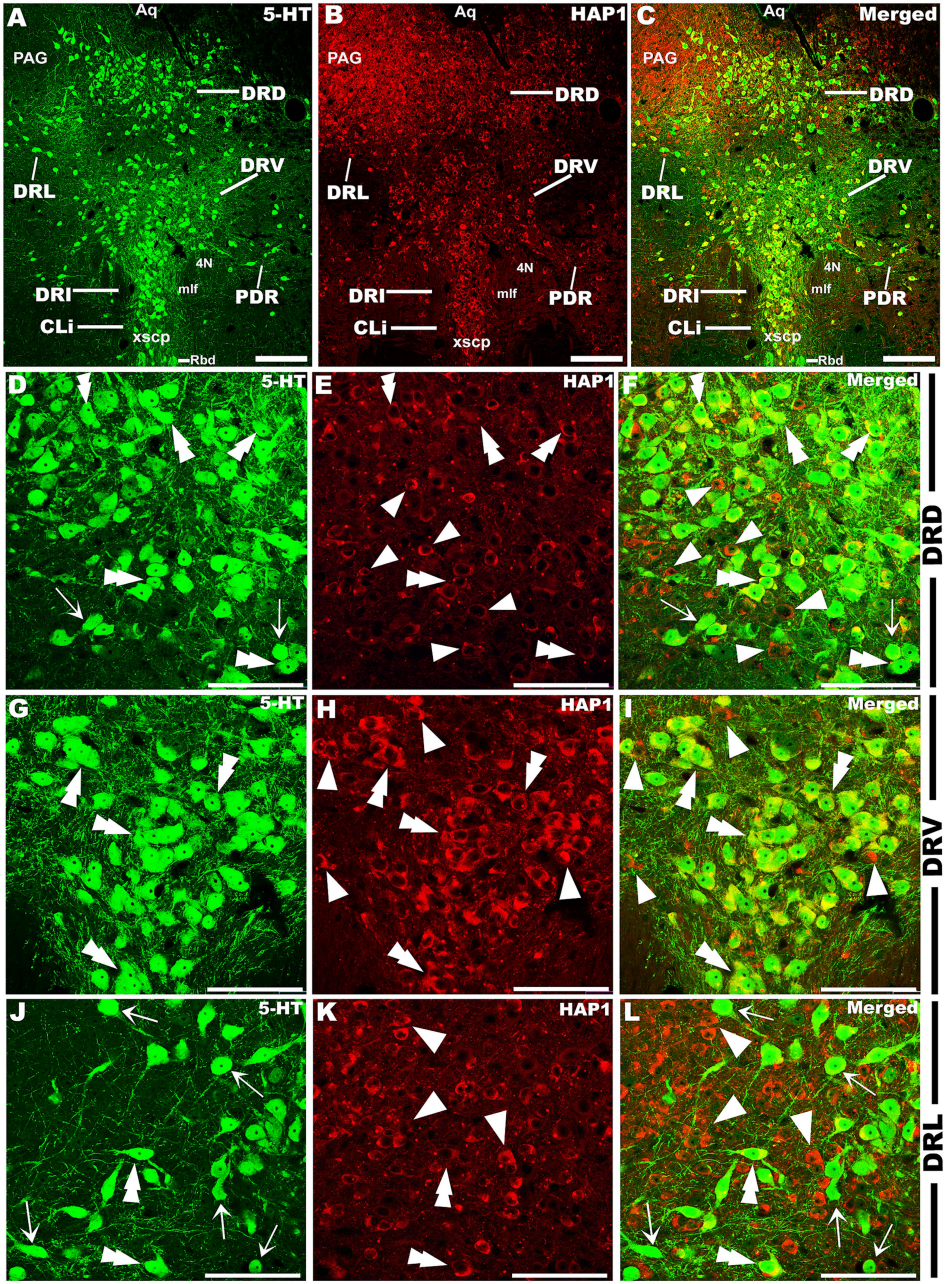
Double-label immunofluorescent immunohistochemistry for 5-HT and HAP1 in the dorsal raphe nucleus at the level of trochlear (4 N) nerve nucleus. Photomicrographs showing double-label immunofluorescence staining of 5-HT and HAP1 in the sub-division of DR **(A–C)**. Panels **(D–L)** are the enlargement of DRD, DRV, and DRL in **(A–C)**. Double arrowheads indicate the cells positive for both HAP1 and 5-HT, white arrows and single white arrowheads indicate the cells positive only for 5-HT and HAP1, respectively. Scale bar = 40 μm in **(A–L)**. For abbreviations, see “List of Glossary”.

**Figure 10:**
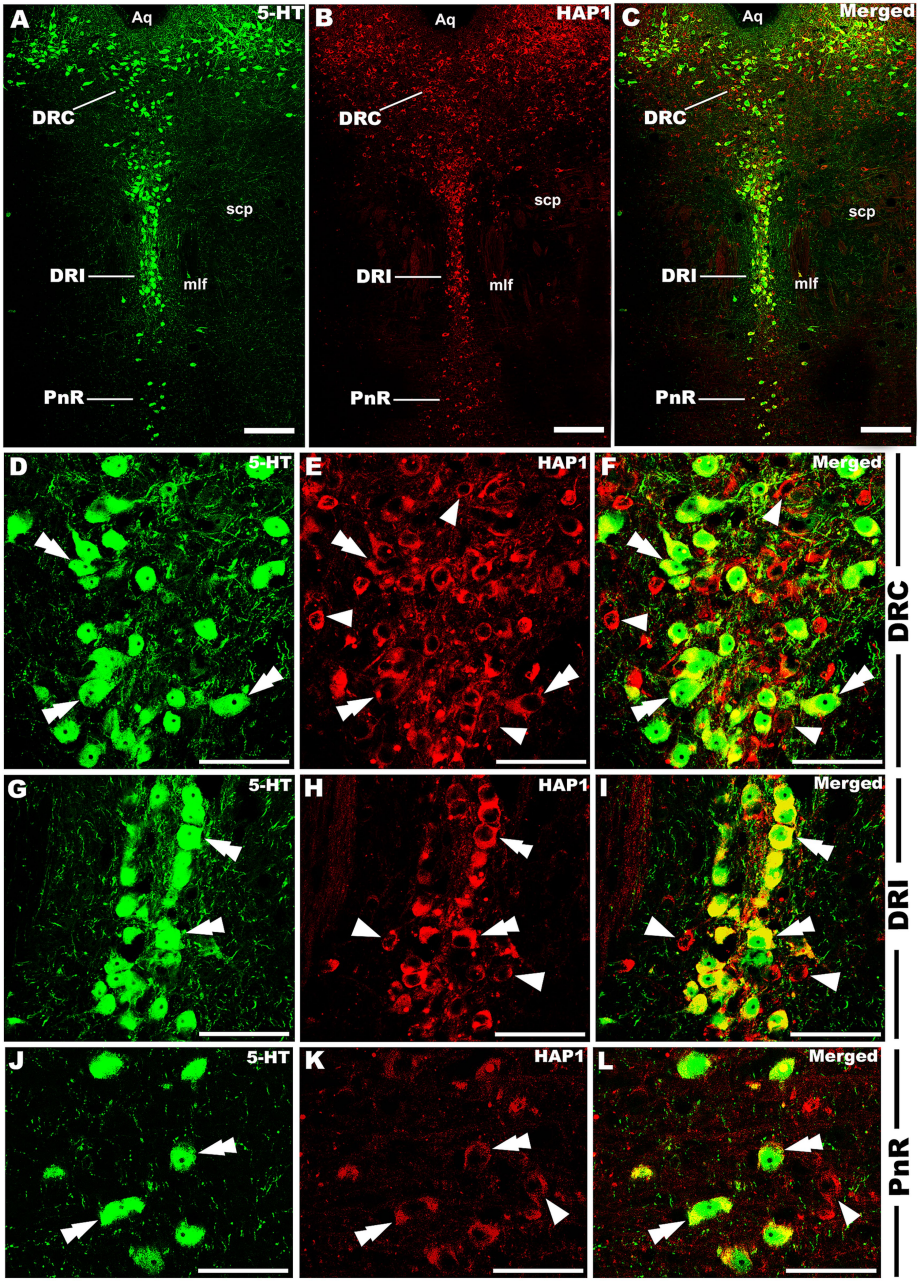
Double-label immunofluorescent immunohistochemistry for 5-HT and HAP1 in the caudal division of the rostral cluster. Photomicrographs showing double-label immunofluorescence staining of 5-HT and HAP1 in the caudal division of the rostral cluster **(A–C)**. Panels **(D–L)** are the enlargement of DRC, DRI, and PnR in **(A–C)**. Double arrowheads indicate the cells positive for HAP1 and 5-HT, white arrows and single white arrowheads indicate the cells positive only for 5-HT and HAP1, respectively. Scale bar = 40 μm in **(A–L)**. For abbreviations, see “List of Glossary”.

**Figure 11:**
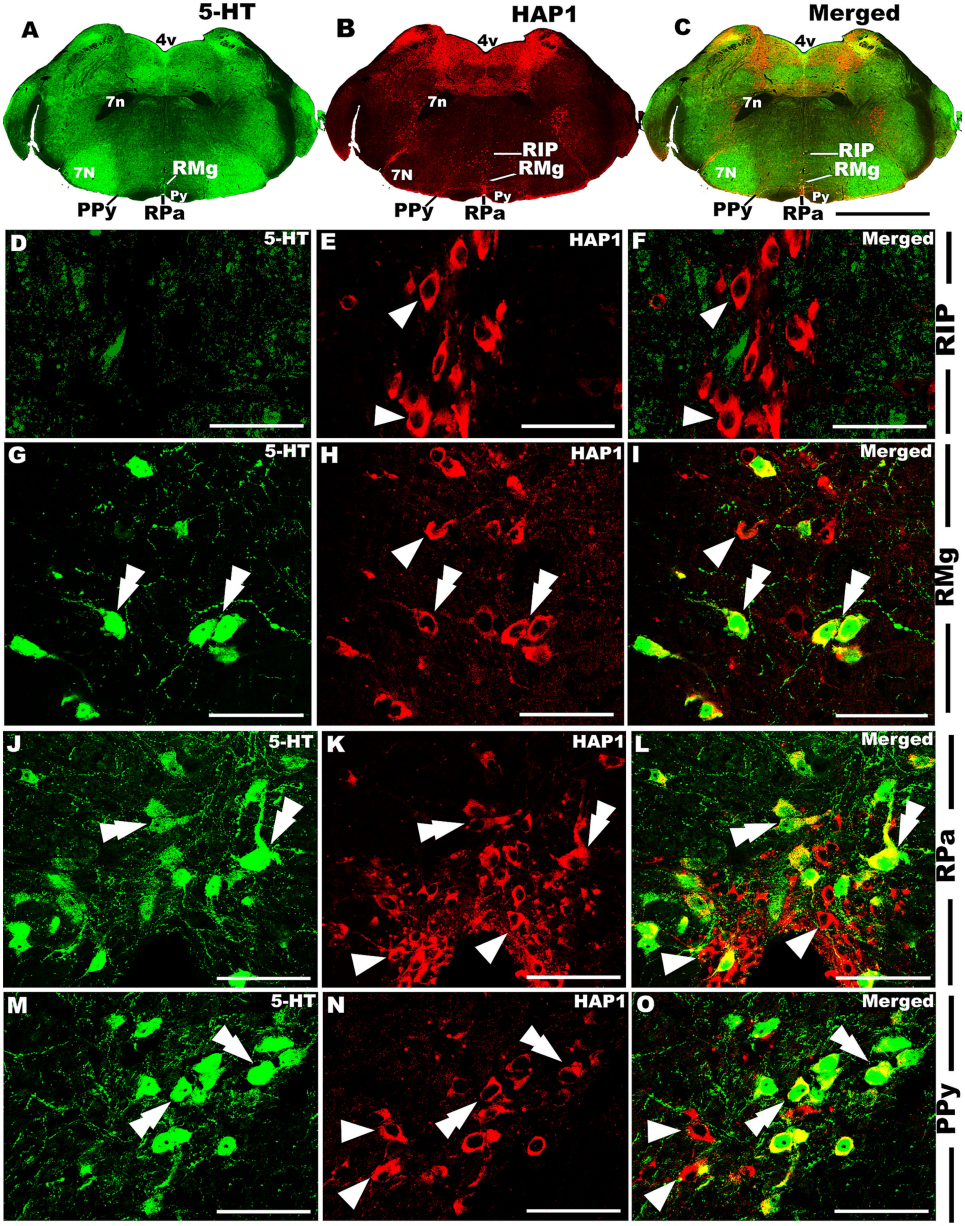
Double-label immunofluorescent immunohistochemistry for 5-HT and HAP1 in the caudal pons. Photomicrographs showing double-label immunofluorescence staining of 5-HT and HAP1 in caudal cluster in caudal pons **(A–C)**. Panels **(D–O)** are the enlargement of RIP, RMg, RPa, and PPy in **(A–C)**. Double arrowheads indicate the cells positive for HAP1 and 5-HT, white arrows and single white arrowheads indicate the cells positive only for 5-HT and HAP1, respectively. Scale bar = 40 μm in **(A–O)**. For abbreviations, see “List of Glossary”.

**Figure 12:**
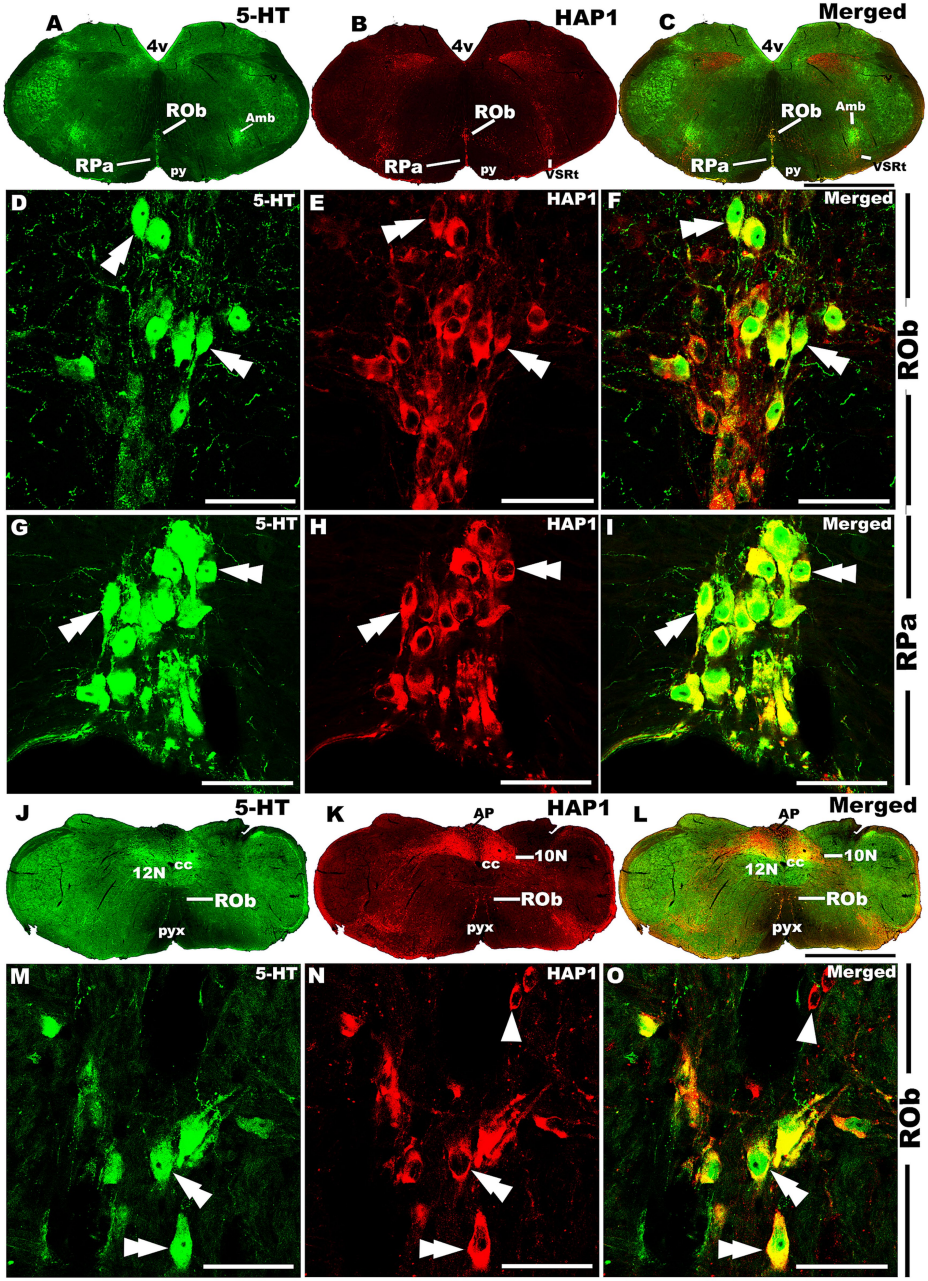
Double-label immunofluorescent immunohistochemistry for 5-HT and HAP1 in the medulla. Photomicrographs showing double-label immunofluorescence staining of 5-HT and HAP1 in caudal cluster in medulla **(A–C,J–L)**. Panels **(D–F,G–I)** are the enlargement of ROb and RPa in **(A–C)**. Panels **(M–O)** is the enlargement of Rob in **(J–L)**, where the Rob gradually splits into two strings. Double arrowheads indicate the cells positive for HAP1 and 5-HT, white arrows and single white arrowheads indicate the cells positive only for 5-HT and HAP1, respectively. Scale bar = 40 μm in **(A–O)**. For abbreviations, see “List of Glossary”.

Our double-labeled immunofluorescence histochemistry indicated that the 5-HT immunopositive cells showed evident HAP1 immunoreactivity in the nuclei of both rostral (Figures 7–10) and caudal clusters (Figures 11, 12), including MnR (Figures 7A–F), SuL (Figures 7A–C,G–L), CLi (Figures 8A–F), PPnR (Figures 8A–C,G–I), five subregions within the DR (Figures 9A–L, 10G–I); DRC (Figures 10A–F); PnR (Figures 10A–C,J–L); RMg (Figures 11A–C,G–I); RPa from caudal pons (Figures 11A–C,J–L) and medulla (Figures 12A–C,G–I) [to which a group of PPy included, Figures 11M–O]; and ROb (Figures 12A–F and upon formation of two parallel vertical laminae Figures 12J–O). Our double-label immunofluorescence staining results, however, confirmed that the HAP1-ir neurons in the mesencephalic PMnR (Figures 7A–C,M–O) and RIP of the caudal pons (Figures 11A–F) never contained 5-HT immunoreactivity.

Our cell counting results showed higher coexpression ratios for HAP1 in 5-HT than those of 5-HT in HAP1 coexpression ratios in most of the rostral and caudal clusters of raphe nuclei, suggesting that the majority of 5-HT neurons contained HAP1 immunoreactivity (Table 3). In midbrain and rostral Pons, CLi exhibited identical HAP1 in 5-HT and 5-HT in HAP1 co-expression ratios of about 97% (Table 3). The DRV also showed high coexpression, with approximately 96.00% of 5-HT-ir neurons containing HAP1 immunoreactivity, while 85.98% of the HAP1-ir neurons had 5-HT immunoreactivity (Table 3). Other nuclei, such as the DRL, exhibited coexpression of HAP1 in 5-HT at 85.65% and of 5-HT in HAP1 at 80.36% (Table 3). However, the MnR displayed a slightly lower coexpression of 5-HT in HAP1 at 70.45%, compared to HAP1 in 5-HT at 95.56% (Table 3).

In the rostral pons, the PnPR exhibited a high coexpression ratio of HAP1 in 5-HT (98.43%) and 5-HT in HAP1 (96.02%). 97.84% of 5-HT-ir neurons showed HAP1 immunoreactivity in the PnR nuclei, while approximately 96.91% of HAP1-ir neurons displayed 5-HT immunoreactivity in that region (Table 3).

The caudal cluster (caudal pons and medulla) of raphe nuclei also exhibited a substantial coexpression ratio of HAP1 with 5-HT. Notably, the RMg had a high coexpression ratio for both HAP1/5-HT (97.42%) and 5-HT/HAP1 (90.68%) (Table 3). In the ROb and RPa, 99.67 and 97.38% of 5-HT neurons contained HAP1 immunoreactivity, respectively. Meanwhile, 94.66% of HAP1-ir cells in ROb and 81.19% of HAP1-ir cells in RPa displayed 5-HT immunoreactivity (Table 3). The RIP, however, stood out by showing no HAP1 coexpression with 5-HT due to a lack of 5-HT neurons (Table 3).

The statistical analyses show significant regional variation in the co-expression ratios of HAP1 and 5-HT throughout the mouse brainstem raphe nuclei. The highest HAP1/5-HT ratios were found in both the rostral and caudal pons and medulla, while fluctuations in co-expression were detected in the midbrain raphe nuclei. Considering inter-animal variation among mice, the significant differences were confirmed by one-way ANOVA followed by Tukey’s test, and specific pairwise comparisons were made using Student’s t-test, showing multiple significant results at both 1 and 5% levels of significance.

## 4 Discussion

The current study, employing Western blot and immunohistochemistry, to the best of our knowledge, represents the first detailed investigation of HAP1 expression and precise distribution in the raphe nuclei throughout the mouse brainstem. As far as we are aware, this study is also the first to map the comparative distribution of HAP1 with 5-HT and their morphological relationships in rostral and caudal clusters of raphe nuclei. The expression of HAP1 in the rodent brainstem has been briefly investigated previously using *in situ* hybridization (Fujinaga et al., 2004) and immunohistochemistry (Islam et al., 2022). However, to our understanding, its cellular localization, comprehensive comparative distribution, and neurochemical characterization in relation to 5-HT have never been investigated in the raphe nuclei.

### 4.1 Comparative distribution of HAP1 and 5-HT-ir neurons in the raphe nuclei

**Figure 13:**
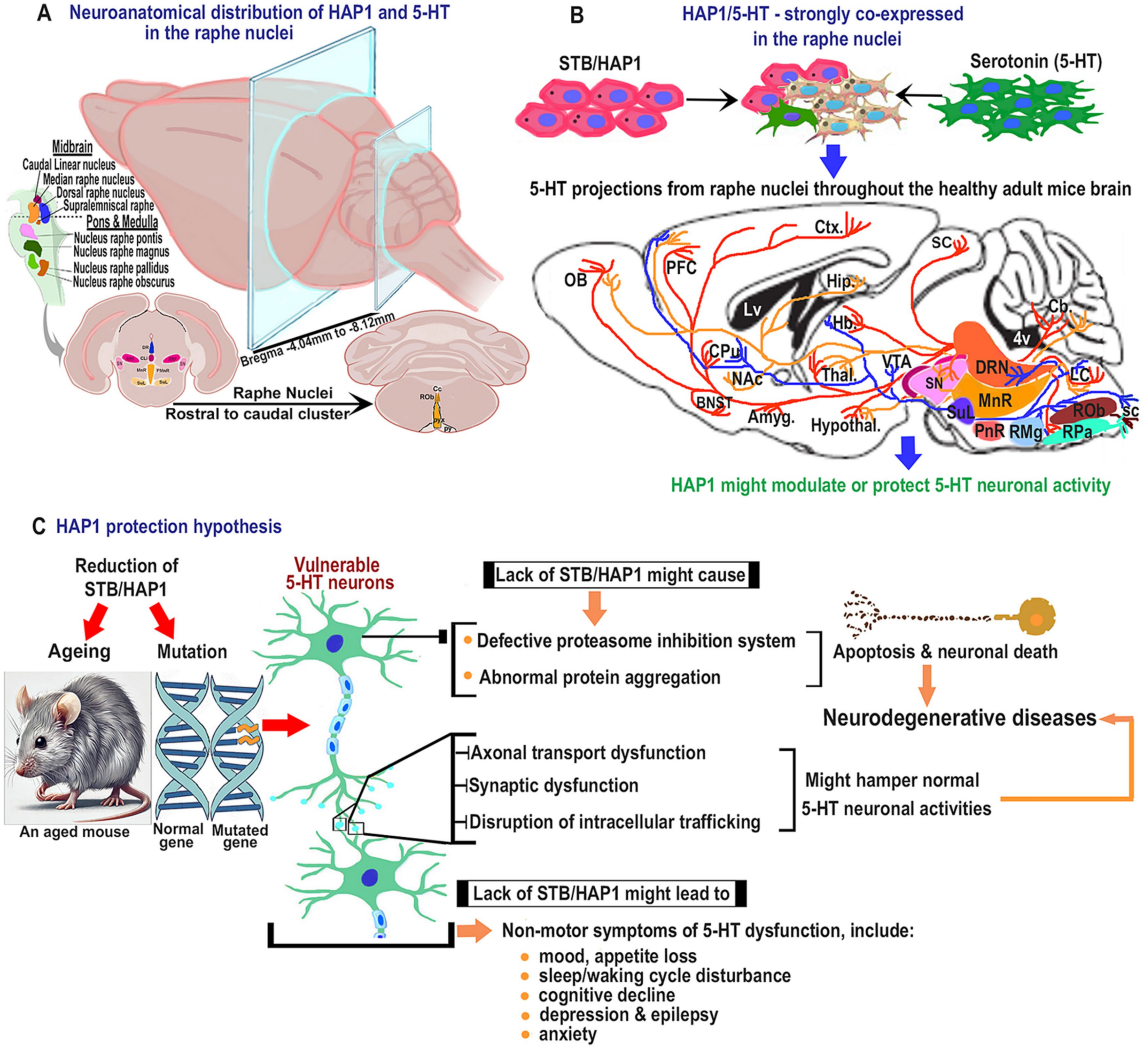
Schematic diagram for the plausible effects of HAP1 in the pathophysiology of serotonin neurons. **(A)** Representative coronal sections for neuroanatomical distribution of HAP1 and 5-HT. **(B)** Illustration of the projections of 5-HT neurons that contain strong HAP1 immunoreactivity in healthy adult mice, which is indicated by the blue arrow. The 5-HT neurons might be protected due to having putative HAP1 protectivity. **(C)** Hypothetical pathways indicate that due to ageing or mutation in HAP1, 5-HT neurons may lack HAP1 protection, which is annotated by the red arrow. The connection between neurodegenerative diseases and the emergence of non-motor symptoms due to 5-HT neuron dysfunction may become more evident in the absence of the putative HAP1 protectivity. Dysfunction of 5-HT neurons and plausible neurodegenerative pathways are indicated by the yellowish arrow. For abbreviations, see “List of Glossary”.

The current study shows that HAP1 immunoreactivity is expressed in the neurons, not glial cells, across the raphe nuclei. These results agree with previous works in other regions of the brain or spinal cord (Islam et al., 2017, 2022; Wroblewski et al., 2018). The midbrain, pons, and medulla have been considered the primary brainstem subdivisions, and serotonergic raphe populations are assigned to these territories (Figure 13A). Our current comparative mapping of HAP1 and 5-HT neurons suggests that both the HAP1 and serotonergic populations present in the rostral cluster essentially belong to different, separate subgroups of nuclei, including CLi, diverse parts of the DR, MnR with ventrolaterally placed SuL, PPnR, and PnR that express high 5-HT immunoreactivity. The PMnR, however, is devoid of 5-HT neurons. The rostral cluster nuclei correspond to the above-mentioned definite brainstem raphe nuclei, as confirmed by prior research (Alonso et al., 2013). Furthermore, five subregions of the DR had been found in rodents by Steinbusch in the early 1980s (Steinbusch, 1981). Our study also offers a more detailed description of the segmental arrangement of the raphe groups, including RMg, ROb, RPa, and medullary PPy, in the caudal cluster that expresses both 5-HT and HAP1. Indeed, our current research clarifies the presence of RIP in the caudal cluster, which shows no 5-HT immunoreactivity but does exhibit HAP1 immunoreactivity. The previous literature (Büttner-Ennever et al., 1988) also aligns with this finding, where RIP lacks 5-HT neurons.

Our earlier research found no apparent sex differences in HAP1 expression in adult mice’s hippocampal region (Islam et al., 2012). Sex variations in the control of serotonergic transmission have been shown in earlier studies, especially in genetically altered models like 5-HT receptor knockout (KO) mice (Jones and Lucki, 2005). Therefore, to completely clarify the cytoarchitectonic and functional variability of HAP1 in the raphe system, additional research, including in-depth investigations of sex as a biological variable across various species, is necessary.

### 4.2 Pathophysiology and possible impact of HAP1 on 5-HT neurons in the raphe nuclei

5-HT neurotransmission generally regulates neurogenesis, synaptogenesis, corticogenesis, neuronal migration, neuronal maturation, and axonal network formation (Ulhaq and Kishida, 2018). Alterations in DR 5-HT neurotransmission have been linked to a number of neuropsychiatric and neurological disorders, including major depressive disorder, bipolar disorder, schizophrenia, obsessive-compulsive disorder, and movement disorders in Parkinson’s disease (PD) (Mahmood and Silverstone, 2001; Vaswani et al., 2003; Huot et al., 2011; Politis and Niccolini, 2015). When it comes to PD, patients’ brains predominantly suffer from increasing dopaminergic denervation, but other systems, such as the 5-HT, are also impacted. 5-HT typically stimulates dopamine release via a range of 5-HT receptors in the normal, non-Parkinsonian brain (Alex and Pehek, 2007). 5-HT activity is generally lower in PD patients, serving as a compensatory strategy for the lower dopaminergic activity (Leentjens et al., 1998). Currently, a number of studies indicate that 5-HT terminal degeneration can cause the development of both motor and non-motor symptoms commonly associated with PD (Halliday et al., 1990; Paulus and Jellinger, 1991; Kish et al., 2008). Braak’s staging describes how the degenerative process in PD progresses gradually upwards (Voon et al., 2006). The rostral raphe nuclei, which house the 5-HT neurons of the brainstem, undergo Lewy body and Lewy neurite deposition during Braak stage II (Braak et al., 2003). Generally, neurodegeneration in the raphe nuclei may be the secondary cause of the loss of striatal 5-HT innervation in PD (D’Amato et al., 1987; Braak et al., 2003). Brainstem serotonergic neurons, however, are anticipated to be affected prior to midbrain dopaminergic neurons in PD. Additionally, a couple of research reveal that when 5-HT level is decreased that cause increased tau expression and cellular aggregation (John et al., 1991) which have been associated with the Alzheimer’s disease (AD) (Yang and Schmitt, 2001; Doraiswamy, 2003; Gudelsky and Yamamoto, 2003; Kovacs et al., 2003).

Besides a robust topographic distribution, our cell counting results suggest that almost all of the 5-HT neurons in raphe nuclei contained STB/HAP1 immunoreactivity (Figure 13B). This implies that HAP1 might modulate 5-HT neuronal activity throughout the healthy brain (Figure 13B). Previous research has suggested that 5-HT, as a neurotransmitter, exerts its effects via membrane receptors in the brain and spinal cord (Hannon and Hoyer, 2008). It is possible that HAP1 could regulate the release of 5-HT from axon terminals by preserving the stability of 5-HT membrane receptors. Intriguingly, it has been reported that membrane receptors cannot be effectively delivered to the proper cellular locations if the association of HAP1 with microtubule-dependent transporters is inhibited (Chen et al., 2023). Additionally, prior studies have shown that HAP1 is connected to synaptic vesicles (Gutekunst et al., 1998) and transfers of different vesicular proteins or membrane receptors, such as vesicles that encompass BDNF (Gauthier et al., 2004). In fractions containing synaptosomes, synaptic vesicle-associated protein (SVP38) and BDNF are reduced in HAP1-KO mouse brain lysate (Lin et al., 2010). HAP1 is particularly crucial for TrkB internalization following BDNF binding (Lim et al., 2018), which is critical for neuron survival in the pathogenesis of AD and PD (Kang et al., 2017). Likewise, synaptic plasticity, neurogenesis, and neuronal survival in the brain are regulated by the 5-HT system, which is acknowledged to interact with the BDNF pathway to create a chain of positive feedback (Mattson et al., 2004). The alteration of vesicular dynamics could be a critical factor in the onset of ND. Hence, it might be postulated that modifying HAP1 expression in 5-HT neurons within the raphe nuclei may cause synaptic disruption, hindering 5-HT neurotransmitter release or neuronal plasticity, which could contribute to neurodegeneration in certain NDs (Figure 13C).

It has been reported that HAP1 can modulate intracellular trafficking (Chen et al., 2023). It is possible that HAP1 could regulate 5-HT neuronal activity via intracellular trafficking, which is crucial for supplying nutrients, molecules, and organelles to nerve terminals. The previous electron microscopy findings demonstrate that HAP1 is dispersed throughout microtubules and membrane-bound organelles (Gutekunst et al., 1998). Consistently, HAP1 may form protein complexes with the trafficking proteins like kinesin light chain and microtubule-dependent transporters, such as dynactin p150, which facilitate the anterograde and retrograde distribution of various cargos or proteins in neuritic processes and nerve terminals (Zhou et al., 2012; Chen et al., 2023; Xiong and Sheng, 2024). It is plausible that a deficiency in HAP1 could lead to a decrease in the quantity of various proteins in the synaptosome-enriched axon terminal, which could potentially impact 5-HT neuronal interactions. Protein mislocalization and the build-up of undegraded proteins in neurons are often caused by abnormalities in intracellular trafficking (Inoshita and Imai, 2015). Therefore, ND pathogenesis may be exacerbated by the reduction in HAP1-mediated intracellular trafficking in 5-HT neurons, especially in long-projecting neurons, which might lead to neuronal dysfunction (Figure 13C). In addition, defective axonal transport of presynaptic cargos or proteins can also cause neurodevelopmental disorders accompanying 5-HT dysfunction, including autism spectrum disorders, attention-deficit/hyperactivity disorder, disruption of learning and motor function, developmental epilepsies, and schizophrenia (Thapar et al., 2017; Sydnor et al., 2021; Xiong and Sheng, 2024). However, identifying serotonergic boutons inside serotonin transporter (SERT) axons utilizing pre- (synaptophysin) and postsynaptic components of excitatory (PSD95) or inhibitory (gephyrin) synapses, and the interaction between HAP1 deficiency and SERT trafficking has yet to be revealed.

Sleep–wake cycle disturbance is the non-motor symptom of PD. The DR is thought to be involved in the sleep–wake cycle control. The activity of the DR is high during waking, low during slow-wave sleep, and abolished during rapid eye movement sleep (Bodner et al., 1995). This notion arises from animal research findings showing that lesions in the 5-HT neurons lead to reduced sleep, which is associated with decreased 5-HT expression in the brain (Bodner et al., 1995) and is responsible for the development of PD. Interestingly, HAP1 is highly expressed in the locus coeruleus, the pedunculopontine nucleus, and the lateral dorsal tegmental nuclei (Islam et al., 2022, 2025). These nuclei are interconnected with the 5-HT neurons from the DR and may play a role in regulating the sleep–wake cycle. Other non-motor symptoms of 5-HT dysfunction include mood disorder, appetite loss, pain, depression, anxiety, fatigue, cognitive decline, hallucinations, sensory and autonomic dysfunction, olfactory deficit, and constipation. Our current immunohistochemical results may indicate that HAP1 possibly controls the above-mentioned non-motor symptoms via modulation of 5-HT neurons in the raphe nuclei (Figure 13C).

Abundant expression of HAP1-ir neurons, other than 5-HT, in PMnR was observed in our study. Neurons of the PMnR are projected to the dorsal horn of the spinal cord and inhibit nociceptive transmission (Millan, 2002). It is noteworthy that our previous studies have demonstrated that HAP1 is highly expressed in the dorsal root ganglion (Islam et al., 2020a) and the superficial layers of the dorsal horn of the spinal cord (Islam et al., 2017). This could either help to inhibit descending pain or modulate specific nociceptive stimuli.

Raphe interpositus in the caudal cluster exhibited HAP1-ir neurons in our current study, which are omnipause neurons (Rüb et al., 2003) and have a potential role in the premotor network for saccades (Langer and Kaneko, 1990). Horizontal saccade slowdown may be a result of the noticeable loss of RIP neurons caused by neurodegeneration in the brainstem of terminal SCA3 patients (Rüb et al., 2003). Through the Josephin domain, HAP1/STB has been shown to interact with both normal and polyglutamine-expanded ataxin-3 mutants (Takeshita et al., 2011). Future studies may focus on how HAP1 alters the physiological function of normal ataxin-3 or how it modifies the toxicity of mutant ataxin-3, influencing the degree of neuronal damage in RIP.

Evidence is mounting that patients with PD, AD, or HD exhibit persistently elevated cortisol levels during stressful situations due to downregulation of glucocorticoid receptor (GR) expression. This suggests that the hypothalamic–pituitary–adrenal (HPA) axis is altered in NDs (Oakley and Cidlowski, 1993; Karanth et al., 1997; Uys et al., 2006; Vyas and Maatouk, 2013; Boero et al., 2018). Another study indicates that a malfunction in 5-HT receptor activity may result from the hypersecretion of cortisol (Pitchot et al., 2001), indicating a reciprocal interaction between the 5-HT and HPA axis. Intriguingly, it has been reported that HAP1 can stabilize the expression of hypothalamic GR (Chen et al., 2020), which is crucial for regulating the HPA axis and alleviating major depression. In addition, the hypothalamus regulates 5-HT neurons through a push-pull mechanism (Zhou et al., 2017), and HAP1 is highly expressed in hypothalamic regions (Fujinaga et al., 2004, 2009). Taken together, our current findings suggest that HAP1 may stabilize 5-HT neurons and enhance or restore 5-HT functions to mitigate depression-like symptoms in PD (Figure 13C).

### 4.3 Plausible HAP1 protective effects in the serotonergic system

Our present research is tempting to speculate that HAP1 might be involved in modulating the serotonergic effects throughout the brain as a “knock-on effect” via the HAP1-expressing 5-HT neuronal projections from the rostral and caudal clusters of the raphe nuclei (Figure 13B). It has been known that HAP1 transfection inhibits neuronal apoptosis triggered by the proteasome inhibitor MG132 in cultured cells (Fujinaga et al., 2011). This highlights the importance of HAP1 in protecting neurons against neurodegenerative apoptosis. It is known that the proteasome inhibition system gradually declines in activity during aging (Zhao et al., 2016). Intriguingly, it has been reported that the expression of HAP1 mRNA is downregulated in the aged rodent brain (Page et al., 1998). Thus, changes in HAP1 expression levels or modifications in the distribution patterns of HAP1 may relate to the reduction in proteasome activity in aged mice brains, which could lead to alterations in HAP1’s protective role against neurodegeneration. Given that HAP1 is also known to be essential for controlling embryonic development, restoring HAP1 expression in neuronal cells throughout developmental stages might prevent early lethality (Dragatsis et al., 2004). In the future, further detailed research should be carried out to examine the HAP1 expression in the raphe nuclei that are subject to temporal regulation throughout the embryonic or aging phases. However, it is intriguing to conjecture that the lower expression of HAP1 immunoreactivity in elderly populations may alter the HAP1 and 5-HT co-expression ratio in the brain, potentially leading to decreased HAP1 protective effects and possibly contributing to neurodegeneration. It is also anticipated that exposure to environmental stress factors may lead to mutations in HAP1, which is likely linked to several NDs due to 5-HT neuronal dysfunctions resulting from changes in HAP1 protectivity (Figure 13C).

### 4.4 Possible effects of HAP1 on other neuronal types: coexist at the raphe nuclei

Though our present study focused on the relationships of HAP1 with 5-HT neurons only, it is well-known that other neuronal types (dopaminergic, peptidergic, GABAergic, and glutamatergic) are present in the raphe nuclei (Huang et al., 2019). 5-HT can regulate the effects of these neurotransmitters in raphe nuclei (Fox et al., 2009). Notably, our current results indicate that the number of HAP1-ir cells might exceed that of 5-HT neurons in the raphe nuclei. Nevertheless, it is possible that HAP1-ir cells found in all raphe nuclei may potentially have an influence on other neuronal types mentioned above. Future studies, however, need to focus on the cytoarchitectonic characterization of HAP1 concerning the dopaminergic, peptidergic, GABAergic, and glutamatergic neurons within specific raphe nuclei.

### 4.5 Limitations of the current study and future perspective

Our current study examined the immunohistochemical relationships between HAP1 and 5-HT in the raphe nuclei of adult male mice. As discussed above, there are potential sex variations in the control of serotonergic transmission; future studies should clarify the relationship between HAP1 and serotonin in female mice across various species. Future research using HAP1-KO mice may shed light on the potential connections between serotonergic neuron growth and functions and the pathophysiology of various NDs. In addition, using animal models that mimic the conditions of certain neurodegenerative diseases (e.g., PD, HD, AD), further study is required to elucidate the precise molecular mechanisms underlying the functional aspects of HAP1, which could be employed in prospective therapeutic applications.

## 5 Conclusion

HAP1 has recently emerged as a neuroprotective binding partner with causal agents of several NDs; however, our neuroanatomical knowledge of HAP1 with 5HT in raphe nuclei remains in its infancy. Our current study reveals the abundant expression of HAP1 immunoreactivity in all the raphe nuclei. This study also clarifies that almost all 5-HT neurons contain HAP1 immunoreactivity in rostral and caudal clusters of raphe nuclei. These findings may provide significant insights into unraveling the role of HAP1 in regulating 5-HT neuronal activity, where HAP1 could act as a neuroprotector that may preserve the integrity of either 5-HT neuron perikarya or its processes.

## Data Availability

The original contributions presented in the study are included in the article/Supplementary material, further inquiries can be directed to the corresponding authors.

## Glossary

2Cb: second cerebellar lobule
3N: oculomotor nucleus
4N: trochlear nucleus
4V: 4th ventricle
5-HT: 5-hydroxytryptamine (serotonin)
5N: trigeminal motor nucleus
6N: abducens nucleus
7N: facial nucleus
7n: facial nerve
10N: dorsal motor nucleus of vagus
12N: nucleus of the hypoglossal nerve
Amb: nucleus ambiguus
AD: Alzheimer’s disease
AP: area postrema
Aq: aqueduct of Sylvius
ATg: anterior tegmental
BDNF: brain-derived neurotrophic factor
cc: central canal
CLi: caudal linear nucleus of raphe
DR: dorsal raphe nuclei
DRC: dorsal raphe nucleus caudal part
DRD: dorsal raphe nucleus dorsal part
DRI: dorsal raphe nucleus interfascicular part
DRL: dorsal raphe nucleus lateral part
DRV: dorsal raphe nucleus ventral part
EW: Edinger-Westphal nucleus
GiA: gigantocellular reticular nucleus
GFAP: glial fibrillary acidic protein
HAP1: huntingtin-associated protein 1
HD: Huntington’s disease
Iba1: ionized calcium binding adaptor molecule 1
IP: interpeduncular nucleus
IPR: interpeduncular nucleus rostral part
IPC: interpeduncular nucleus caudal part
ir: immunoreactive
ml: medial lemniscus
mlf: medial longitudinal fasciculus
MnR: median raphe nucleus
NDs: neurodegenerative disorders
NeuN: neuronal nuclei
PAG: periaqueductal gray
PD: Parkinson’s disease
PDR: posterodorsal raphe
PMnR: paramedian raphe nucleus
PPnR: prepontine raphe nucleus
PnC: pontine reticular nucleus caudal part
PnR: pontine raphe nucleus
PnO: pontine reticular nucleus oral part
PPy: parapyramidal nucleus
py: parapyramidal tract
r1: rhombomere 1
r2: rhombomere 2
r3: pontine rhombomere 3
R: red nucleus
Rbd: rhabdoid nu
RIP: raphe interpositus nucleus
RMg: raphe magnus nucleus
ROb: raphe obscurus nucleus
RPa: raphe pallidus nucleus
RRF: retrorubral field
RRID: research resource identifier
scp: superior cerebellar peduncle
sp5: spinal trigeminal tract
STB: stigmoid body
SuL: supralemniscal raphe nucleus
TBST: tris-buffered saline with 0.1% Tween 20
TrkB: tropomyosin receptor kinase B
ts: tectospinal tract
VSRt: ventrolateral superficial reticular area
xscp: decussation of the superior cerebellar peduncle
xpy: pyramidal decussation
