# Supplementary material for: Neuroanatomical mapping of huntingtin-associated protein 1 across the rostral and caudal clusters of mouse raphe nuclei and its immunohistochemical relationships with serotonin
Source: Front Neuroanat. 2025 Jul 22;19:1625793. doi: 10.3389/fnana.2025.1625793 (PMC12321798; doi:10.3389/fnana.2025.1625793)
Supplement: Supplementary file 1 [file Data_Sheet_1.PDF]

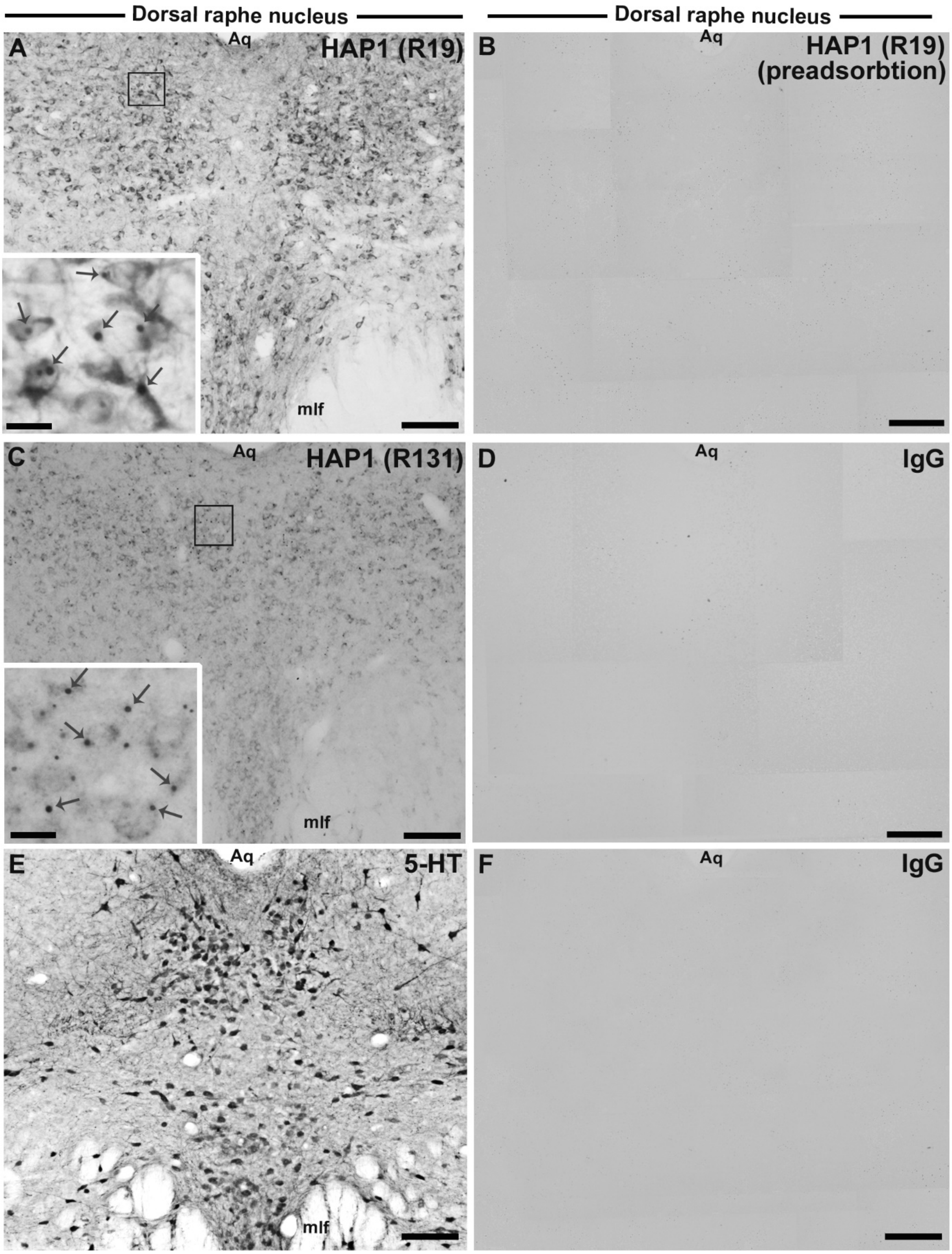

Figure 1. Immunoperoxidase immunohistochemistry in the dorsal raphe nucleus. (A, C) HAP1 immunoreaction throughout the dorsal raphe nucleus using R19 and R131 antibodies, respectively. Black arrows indicate the HAP1-ir stigmoid body (STB) in the insets of A and C. (B) Pre-adsorption of the anti-HAP1 R19 antibody with a blocking peptide eliminates the HAP1 immunoreactivity. (E) Expression of 5-HT neurons within the dorsal raphe nucleus using anti-5-HT antibody. (D, F) Normal rabbit IgG resulted in the disappearance of immunoreactivity in the dorsal raphe nucleus. Scale bar = 20 μm in A-F and insets. For abbreviations, see *Glossary.*
